# Prefrontal tDCS attenuates counterfactual thinking in female individuals prone to self-critical rumination

**DOI:** 10.1038/s41598-021-90677-7

**Published:** 2021-06-02

**Authors:** Jens Allaert, Rudi De Raedt, Frederik M. van der Veen, Chris Baeken, Marie-Anne Vanderhasselt

**Affiliations:** 1 Department of Head and Skin, Ghent University, University Hospital Ghent (UZ Ghent), 1K12F, Corneel Heymanslaan 10, 9000 Ghent, Belgium; 2 Ghent Experimental Psychiatry (GHEP) Lab, Ghent University, Ghent, Belgium; 3 Department of Experimental Clinical and Health Psychology, Ghent University, Ghent, Belgium; 4 Department of Psychology, Education and Child Studies, Erasmus University Rotterdam, Rotterdam, The Netherlands; 5 Department of Psychiatry, Vrije Universiteit Brussel (VUB), University Hospital UZBrussel, Brussels, Belgium

**Keywords:** Physiology, Psychology, Risk factors

## Abstract

The tendency to ruminate (i.e., repetitive negative self-referential thoughts that perpetuate depressive mood) is associated with (a) an elevated propensity to maladaptively experience counterfactual thinking (CFT) and regret, and (b) hypo-activity of the left dorsolateral prefrontal cortex (DLPFC). The goal of this study was to investigate whether anodal transcranial direct current stimulation (tDCS) over the left DLPFC, in function of self-critical rumination tendencies, momentarily reduces counterfactual thinking and regret (assessed via self-report and psychophysiological indices). Eighty healthy participants with different levels of self-critical rumination received either anodal or sham tDCS while performing a decision making task in which they were repeatedly confronted with optimal, suboptimal, and non-optimal choice outcomes. The results showed that among rumination-prone individuals, anodal (versus sham) tDCS was associated with decreased CFT and attenuated psychophysiological reactivity to the differential choice outcomes. Conversely, among low rumination-prone individuals, anodal (versus sham) tDCS was associated with increased CFT and regret, but in absence of any effects on psychophysiological reactivity. Potential working mechanisms for these differential tDCS effects are discussed. Taken together, these results provide initial converging evidence for the adaptive effects of left prefrontal tDCS on CFT and regret to personal choice outcomes among individuals prone to engage in self-critical rumination.

## Introduction

Feelings of contentment regarding one’s own life trajectory, and the personal choices made here in, play an important role in the development and maintenance of positive mental health outcomes^1,2^. Past research has shown that individuals prone to ruminate are more likely to respond negatively to past personal choice outcomes^3–6^, with rumination being a well-known vulnerability factor for depression and referring to repetitive negative self-referential cognitions and emotions^7^. For instance, when individuals are confronted with progress towards a personal goal that is either blocked (i.e., non-optimal) or unsatisfactory (i.e., suboptimal), they may, in varying degree, contemplate about the possible outcomes of alternative choices in the past, referred to as counterfactual thinking (CFT), and experience self-blame regret^3,8–11^. These responses are most likely to occur when individuals have the perception that another personal, incorrigible decision in the past might have led to more favorable results (lost opportunities^12^). Whereas these responses are thought to serve the adaptive purpose to improve future decision making^9^, the excessive experience of CFT and regret has been consistently shown to be positively associated with depressive symptomatology and negatively with life satisfaction and subjective well-being^2,13,14^. Therefore, in light of these mental health implications^15^, it is of crucial importance to investigate potential interventions to attenuate these responses to personal decision making outcomes, specifically in populations that are prone to respond maladaptively, such as rumination-prone individuals^3,7^.

One way to potentially reduce these maladaptive responses in individuals prone to ruminate, is to influence brain areas and neural networks that are involved in the processes underlying rumination, by means of non-invasive brain stimulation (NIBS^16,17^). Based on neuro-imaging studies in both healthy and depressed populations, the tendency to ruminate has been shown to be associated with hypo-activation of the left prefrontal cortex, which is thought to reflect reduced cognitive control over (negative) emotional information, such as the ability to exert influence over the experience of emotional states^17–20^. The dorsolateral prefrontal cortex (DLPFC) is an important hub in the implementation of (emotional) cognitive control processes^21,22^, and is a common target within NIBS research and interventions^23,24^. For instance, research has shown that anodal transcranial direct current stimulation (tDCS) over the left prefrontal cortex, thought to modulate underlying neuronal activity, can momentarily improve cognitive control, diminish ruminative processes, reduce emotional reactivity, and stimulate adaptive emotion and stress regulatory processes^25–29^. tDCS is a low-cost, easy to use technique, and is self-administrable, thereby showing essential characteristics for a potential feasible intervention^30,31^. Moreover, left prefrontal tDCS shows some promise as an intervention to treat affective disorders^32,33^. Despite these findings, the effect sizes of NIBS interventions are modest at best (for a meta-analysis, see Smits et al.^29^). However, there is growing evidence that individual differences play a pivotal role in their efficacy^34–37^. For instance, in a healthy population, it has been shown that only individuals with high habitual rumination tendencies display beneficial effects of NIBS on stress regulation^38^. Thus, it could be expected that individuals with high ruminative predispositions might especially benefit from left prefrontal anodal tDCS, as this is thought to modulate left hemispheric hypo-activity and restore the hemispheric imbalance that is characteristic of this population^18,39,40^.

In view of the mental health implications associated with excessive CFT and regret^3,7,13,14^, the goal of this study was to investigate whether anodal tDCS over the left DLPFC could momentarily attenuate CFT and regret. Based on the premise that rumination-prone individuals are more prone to excessively experience CFT and regret, and display hypo-activation of the left prefrontal cortex^18,39,40^, we hypothesized that anodal (versus sham) tDCS over the left DLPFC—thought to modulate underlying neuronal activity—would diminish CFT and regret specifically in this population, as compared to individuals with low ruminative tendencies. To test this, we employed a validated paradigm in which a self-relevant decision making context is simulated, and where CFT and regret are naturally triggered in varying degree among participants with differing levels of rumination tendencies^1,3,27^. In this paradigm, participants repeatedly make choices that are ultimately linked to the goal of obtaining a monetary reward, while under the impression that their task performance is predictive of self-relevant life outcomes, such as mental health, career success, and relationships. After each set of choices, participants are presented with the outcomes of these choices relative to the outcomes of alternative, counterfactual choices. The specific combination of these informs participants about the relative utility of these choices towards their goal progress (i.e., non-optimal, suboptimal, optimal). To measure indices of CFT and regret in this context, both self-report and psychophysiological assessments (skin conductance and heart rate) were utilized^1^. Skin conductance responses (SCRs) reflect an indirect marker of sympathetic autonomic activity, with higher SCRs being indicative of larger emotional arousal, and vice versa^1,41,42^. Heart rate (HR) informs about the temporal dynamics of cardiovascular reactivity to stimuli, and is the result of the concurrent interaction between sympathetic acceleratory and parasympathetic deceleratory influences of the autonomic nervous system^43^. It has been shown that tasks that predominantly rely on information processing and cognitive elaboration, such as decision making and logical reasoning, often elicit HR accelerations^1,44,45^, where the level of cognitive engagement is positively associated with HR acceleration^46–48^. On the other hand, tasks that mainly rely on attention to external stimuli, such as perception of (self-relevant) emotional stimuli, typically produce HR decelerations as a function of perceived threat^49–51^. Because recent symptom network approaches suggest that self-critical cognitions play a central role in rumination^52,53^, and the inherent self-referential nature of CFT and self-blame regret, we chose to operationalize rumination in the form of self-critical rumination, as this construct emphasizes on self-critical aspects of repetitive negative thought^54^. Moreover, because it has been consistently shown that women display greater rumination propensities, (for a meta-analysis, see Johnson et al.^55^), to reduce variability, and thereby increase statistical power, the current study focused exclusively on females.

In summary, the goal of this study was to (a) investigate whether, as a function of tendencies towards self-critical rumination, anodal tDCS over the left DLPFC momentarily attenuates CFT and regret, and (b) to explore psychophysiological correlates of this hypothesized tDCS effect in response to varying choice outcomes related to goal progress (i.e., non-optimal, suboptimal, optimal).

## Materials and methods

### Participants

Eighty healthy female individuals (*M*_age_ = 21.06, *SD*_age_ = 1.93) from the general community were recruited via internet postings on social media and posters in public places. An a priori power analysis in G*Power^56^ estimated a total sample size of n = 77 to detect with 80% power a moderate to large (Cohen’s f = 0.325) interaction effect for an ANCOVA f-test. This was rounded up to n = 80 to have a margin (3.75%) for potential data loss. Selection criteria were in line with our prior tDCS studies^27,57^: (a) normal or corrected to normal vision, (b) no current psychiatric or neurological disorders, (c) no current use of psychiatric drugs, (d) no personal or family history of epilepsy, (e) no history of serious head injury or neurosurgery, (f) not pregnant, and (g) no metal or magnetic objects in or around the scalp, (h) no participation in more than ten psychological experiments, and (i) right handed. Participants were asked not to smoke or ingest caffeine and/or alcohol 2 h prior to the experiment. The study was conducted with the approval of Ghent University’s Medical Ethical Committee, and participants’ informed consent was obtained according to the Declaration of Helsinki. The experiment was performed in accordance with relevant guidelines and regulations. Participants received € 17 for participating.

### Materials

#### Devil’s task

**Figure 1:**
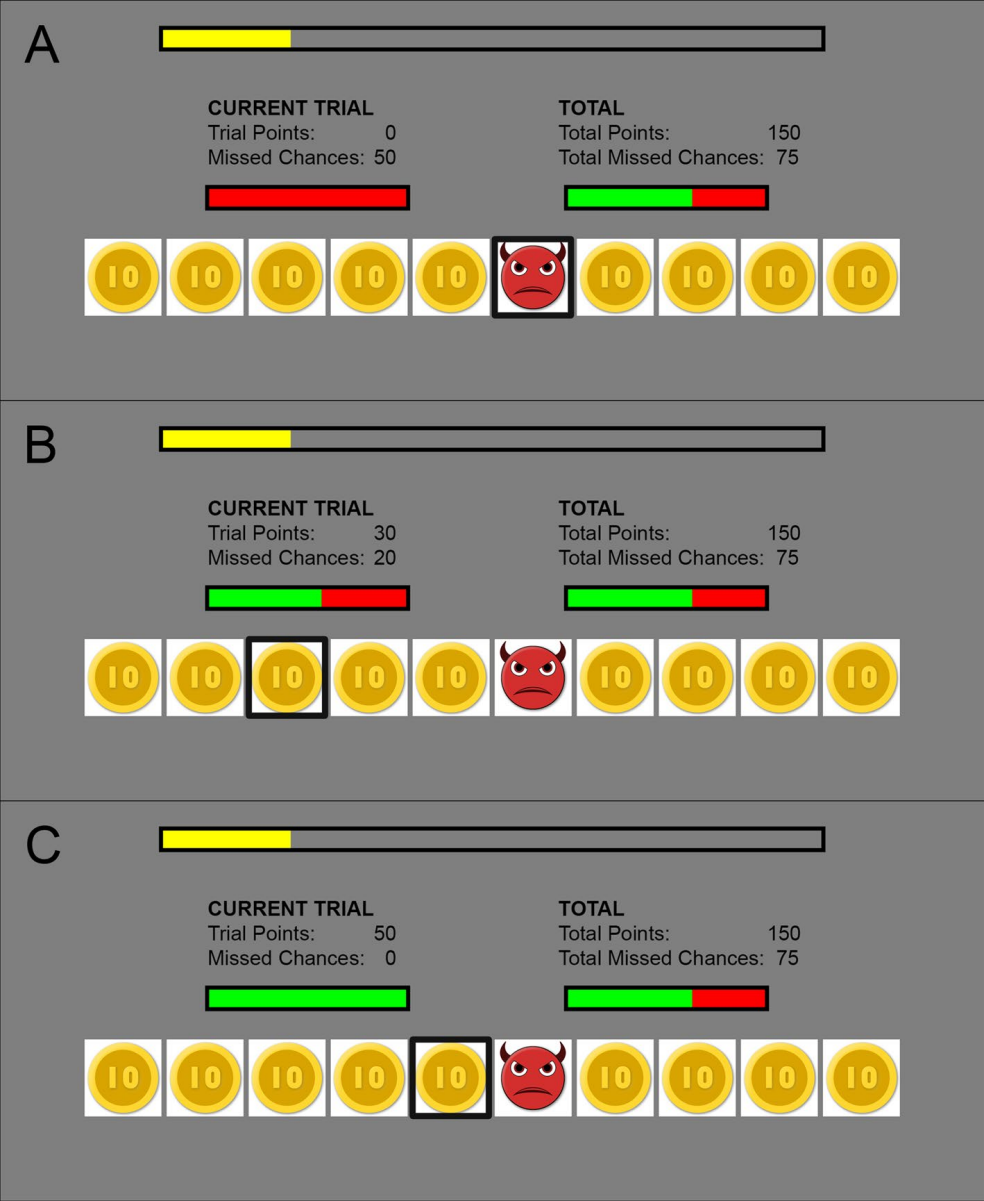
Choice outcome display at the end of a trial. Due to copyright restrictions, the presented stimuli depicting points and the devil slightly differ from the actual employed stimuli in the paradigm.

An adaption of the Devil’s task, a sequential binary choice risk-taking paradigm, was used^3,58^. Participants were presented an information display telling them they would perform a choice task that has been found to be predictive for various life domains, such as academic success, interpersonal skills, coping behavior, and psychopathology, and that they could obtain a monetary bonus if they performed well enough. This information was merely presented as a cover story, to simulate a self-relevant decision making context^3^. Participants were told to try to accumulate as much points as possible in order to obtain the monetary reward, and that their progress towards this reward could be monitored throughout the task by means of a progress bar. During each trial, ten closed boxes were presented, from which nine contained 10 points, whereas 1 contained a ‘devil’. The task consisted of 100 trials, and the devil’s location was pseudorandomized so that the devil appears equally in every box. Participants could open one box at a time, in a left to right fashion. If a box was opened that contained the ‘devil’, all trial points were lost. To consolidate trial points, participants can choose when to stop opening boxes. At the end of every trial (by either stopping or unpacking the devil), the contents of all boxes were revealed and a score bar indicated factual versus counterfactual outcomes, for a period of 8 s to allow for SCRs to return to baseline^59^. During this reveal (Fig. 1), participants perceive the possible discrepancy between factual and counterfactual outcomes, indicative of the utility of their choice outcomes. These outcomes can be classified into three categories: (a) non-optimal, referring to lost opportunities with no factual gains, indicative of no goal progress, (b) suboptimal, referring to factual gains and lost opportunities, indicative of some goal progress, and (c) optimal, referring to factual gains with no lost opportunities, indicative of optimal goal progress. After every four trials, a break period was introduced, during which participants were visually presented factual versus counterfactual outcomes of the last four trials, and throughout the entire task. At that point, participants self-reported CFT and regret. These self-report measures were only done every four trials to prevent fatigue among participants and to retain a natural flow in the task. At the end of the task, participants were shown via the progress bar that they did not perform well enough in order to obtain the monetary reward. This was done by setting the point threshold to completely fill the progress bar to an amount (2600) that was slightly above the points that would be obtained when using the optimal strategy of consistently opening half of the boxes (2500). The paradigm was programmed in MATLAB 2018b (The MathWorks Inc,), using the psychtoolbox^60^.

#### Transcranial direct current stimulation (tDCS)

**Figure 2:**
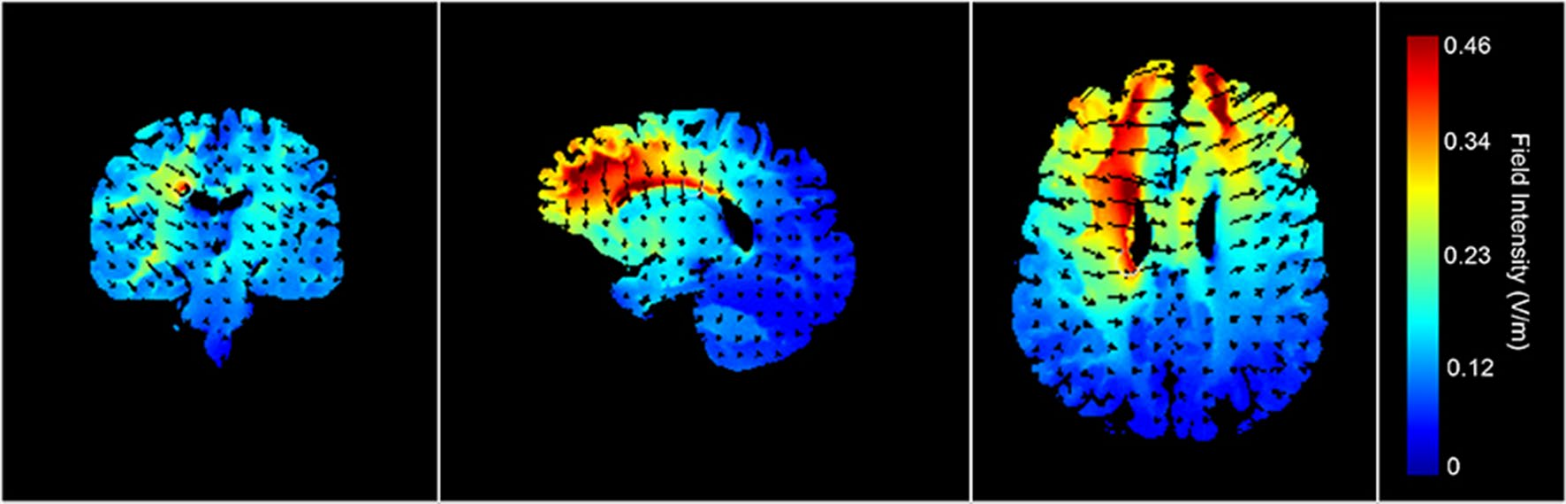
Electric field simulations of the tDCS montage.

TDCS was applied with a pair of saline-soaked sponge electrodes (5 × 7 cm = 35 cm^2^), and delivered with a battery-driven stimulator (1 × 1 tDCS mini-CT, Soterix Medical Inc.) The anodal electrode was vertically positioned over F3 (corresponding to the left DLPFC) according to the 10–20 international EEG system, whereas the cathode was placed over the contralateral supra-orbital area (Fp2). This electrode positioning is in accordance with previous tDCS studies on emotional processing and the level B recommendation for treating major depressive disorders^23,61,62^. A current of 2 mA (current density = 0.06), with 30 s of ramp up was applied for 20 min with a 30 s ramp down at the end. 50% of the participants received active anodal tDCS, whereas the others received sham tDCS (between-subject design). For sham tDCS (i.e., placebo) the current was directly ramped down after the initial ramp up phase^62^. The tDCS procedure followed a single-blind design. Figure 2 shows a visualization of the electric field simulation of the utilized tDCS montage, using Soterix HD-Explore software.

#### Self-report measures

##### Online survey

To ensure comparable anodal and sham tDCS groups, an online survey assessing potential confounders (e.g., trait regret proneness, behavioral inhibition and reward sensitivity, symptoms of mood and anxiety disorders, and the habitual use of adaptive and maladaptive emotion regulation strategies) was carried out prior to the experiment. Independent t-tests (Supplementary Table S1) showed no group differences on any of these variables (all *p*s > 0.17).

##### Self-critical rumination

The habitual tendency to engage in self-critical rumination (e.g., “I often worry about all the mistakes I have made”, “Sometimes it is hard for me to shut off critical thoughts about myself”) was assessed using the Self-Critical Rumination Scale (SCRS^54^). The scale consists of ten items, rated on a 4-point (1 = not at all, 2 = a little, 3 = moderately, 4 = very much) Likert scale. Participants were asked to indicate how well each item described them. The scale displayed excellent internal consistency (Cronbach’s α = 0.92).

##### Counterfactual thinking and regret

Counterfactual thinking and regret were assessed at the end of every four trials (i.e., during the task), and at the end of the task, when participants knew they did not perform well enough for the monetary reward (post-task; see “Supplementary Materials”). Participants were displayed “To what extent do you think about what others choices would have led to?” and “To what extent do you regret the made choices?”, and responded using a visual analog scale (VAS), ranging from 0 = not at all, to 100 = a lot.

#### Psychophysiological measures

##### Skin conductance responses (SCRs)

Electrodermal activity was recorded at a 1000 Hz sample rate with the Biopac EDA100c amplifier, in conjunction with the Biopac MP150 (Biopac Systems Inc., Santa Barbara, CA). On the amplifier, the gain was set to 5 μS/V, the low pass filter set to 10 Hz, and both high pass filters set to DC mode (off). Gelled velcro finger electrodes were placed on the distal phalanges of the index and middle finger of the non-dominant (left) hand^63–65^. The data was collected in the Acqknowledge software on an external computer, together with event triggers that were sent by the MATLAB computer to the Biopac STP100c, via a USB interface. Using the LedaLab 3.4.9 MATLAB toolbox^66^, the data was downsampled to 50 Hz (to increase processing speed), artifact corrected using spline interpolation, smoothed using a 16 sample moving average, and filtered using a 1st order Butterworth 5 Hz lowpass filter. Continuous decomposition analysis then extracted the phasic information from the skin conductance signals, based on a SCR detection threshold of 0.01 µS^67^, and computed average SCR amplitude (expressed in µS) for every 8 s outcome reveal at the end of each trial.

##### Cardiovascular reactivity

Cardiovascular reactivity was recorded at a 1000 Hz sample rate with the Biopac ECG100c amplifier, in conjunction with the Biopac MP150 (Biopac Systems Inc., Santa Barbara, CA). On the amplifier, the gain was set to 5000, mode set to normal, low pass filter set to 35 Hz and the high pass filter set to 0.05 Hz. Pre-gelled Ag/AgCL electrodes were placed according the measurement of a lead II ECG. The data was collected in the Acqknowledge software on an external computer, together with event triggers that were sent by the MATLAB computer to the Biopac STP100c, via a USB interface. Using the PhysioData Toolbox 0.4^68^, the signal was first filtered using a 1 Hz highpass filter and a 50 Hz lowpass filter, and R-peaks and interbeat intervals (IBIs) were subsequently automatically detected based on the following constraints: (a) minimum R-peak of 0.5 mV, (b) minimum IBI of 0.3 s, and (c) maximum IBI of 1.5 s. Based on visual signal inspection, artifacts in the R-peaks and IBIs were then removed and interpolated if possible. Next, a continuous heart rate signal was computed via shape-persevering piecewise cubic interpolation at 100 Hz of the valid IBI data. Based on this signal, HR was computed for every second during the 8 s outcome reveal, and 1 s prior to the outcome reveal (i.e., pre-outcome baseline). The change in HR (expressed in bpm) was then computed for every second during the 8 s outcome reveal, by subtracting HR during the pre-outcome baseline from the HR values at every second of the outcome reveal^1^.

### Procedure

Before the lab session, on an online webpage participants read a description of the study, including the exclusion criteria and part of the cover story. To mask the true intent of the study, participants were made to believe they were participating in a study investigating the effects of tDCS on decision making behavior. In addition, they read an information form explaining (a) that tDCS induces a weak electrical current flowing from the anode to the cathode, through underlying brain regions, which thereby modulates their neural activity, and (b) the safety and potential short-term side effects (e.g., headache, dizziness, nausea) of tDCS. They were informed they would either receive active or sham tDCS during the lab session. Afterwards, they completed the online survey and based on this survey data, participants were pseudo-randomly (see “Supplementary Materials”) assigned to either the anodal or sham tDCS group. At the start of the lab session, participants provided written informed consent. Participants were seated in front of a computer screen and were connected to the physiological recording equipment. Next, anodal or sham tDCS was administrated while participants were introduced to the task and subsequently performed it. At the end of the task, participants were compensated and verbally debriefed: the cover story and the nature of the regret-inducing paradigm was explained. The duration of the protocol was on average 40 min.

### Data analytic plan

All data was analyzed in R 3.6.1^69^ in conjunction with Rstudio 1.2.1335. Analyses were conducted by either fitting linear mixed models (LMMs) or generalized linear mixed models (GLMMs), depending on the distribution (i.e., normal or gamma) of the outcome variable (see “Supplementary Materials”). These models were fitted via the ‘lmer’ and ‘glmer’ functions of the ‘*lme4*’ and ‘*lmertest*’ R packages^70,71^. The sum of squares for the models were estimated using the type III approach, and the statistical significance level was set to *p* < 0.05. Continuous predictors were standardized prior to model fitting. For the decomposition of interaction effects, follow-up tests were either pairwise comparisons of the estimated marginal means (EMMs) or pairwise comparisons of the EMMs of linear trends (i.e., comparisons of slopes), via the ‘emmeans’ and ‘emtrends’ function in the *‘emmeans’* R package^72^. For interaction effects were self-critical rumination was implied, these follow-up tests were carried out at different levels of self-critical rumination, by computing the EMMs at three values (*M* − 1 *SD* [low], *M* [moderate], *M* + 1 *SD* [high]) of self-critical rumination. This procedure preserves the continuous measurement structure of the self-critical rumination variable and mitigates loss of statistical power present in often employed procedures in which continuous variables are categorized into groups^73^. Where applicable, *p*-values from follow-up tests were corrected for multiple comparisons using the false discovery rate correction^74,75^.

First, to investigate the role of self-critical rumination on effects of tDCS on reported CFT and regret during the task, 2 LMMs were fitted, featuring CFT and regret every four trials during the task as dependent variables, and *group* (sham tDCS, anodal tDCS) as fixed factor, *recent lost opportunities* (i.e., lost opportunities of the last four trials) and *self-critical rumination* as continuous predictors, and *subject* as random intercept.

Second, to investigate the role of self-critical rumination on effects of tDCS on SCRs and cardiovascular reactivity to trial-based choice outcomes, a GLMM for a gamma distribution with a log-link function was fitted with the amplitude of the skin conductance response as dependent variable, whereas a LMM was fitted with the change in heart rate as dependent variable. Both these models featured *group* (sham tDCS, anodal tDCS), *choice outcome* (non-optimal, suboptimal, loss) as fixed factors, *self-critical rumination* as continuous predictor, and *subject* as random intercept. The LMM for the cardiovascular reactivity additionally featured *time* (i.e., elapsed seconds after choice outcome; 1, 2, 3, 4, 5, 6, 7, 8) as fixed factor.

The effects where *group* or *choice outcome* (for psychophysiological analyses) were not implied, are reported in “Supplementary Materials” for brevity, as these fall outside the scope of our research aims.

## Results

Participants were not able to correctly gauge their stimulation group (sham, anodal tDCS), as the proportion of incorrect guesses (63%) was higher than change level (50%), *p* = 0.03. Furthermore, there were no significant differences in the time of day during the experimental sessions between the the anodal and sham tDCS group, *t* = 0.93, *p* = 0.36.

### Effects of tDCS on self-reported counterfactual thinking and regret

#### Counterfactual thinking

**Figure 3:**
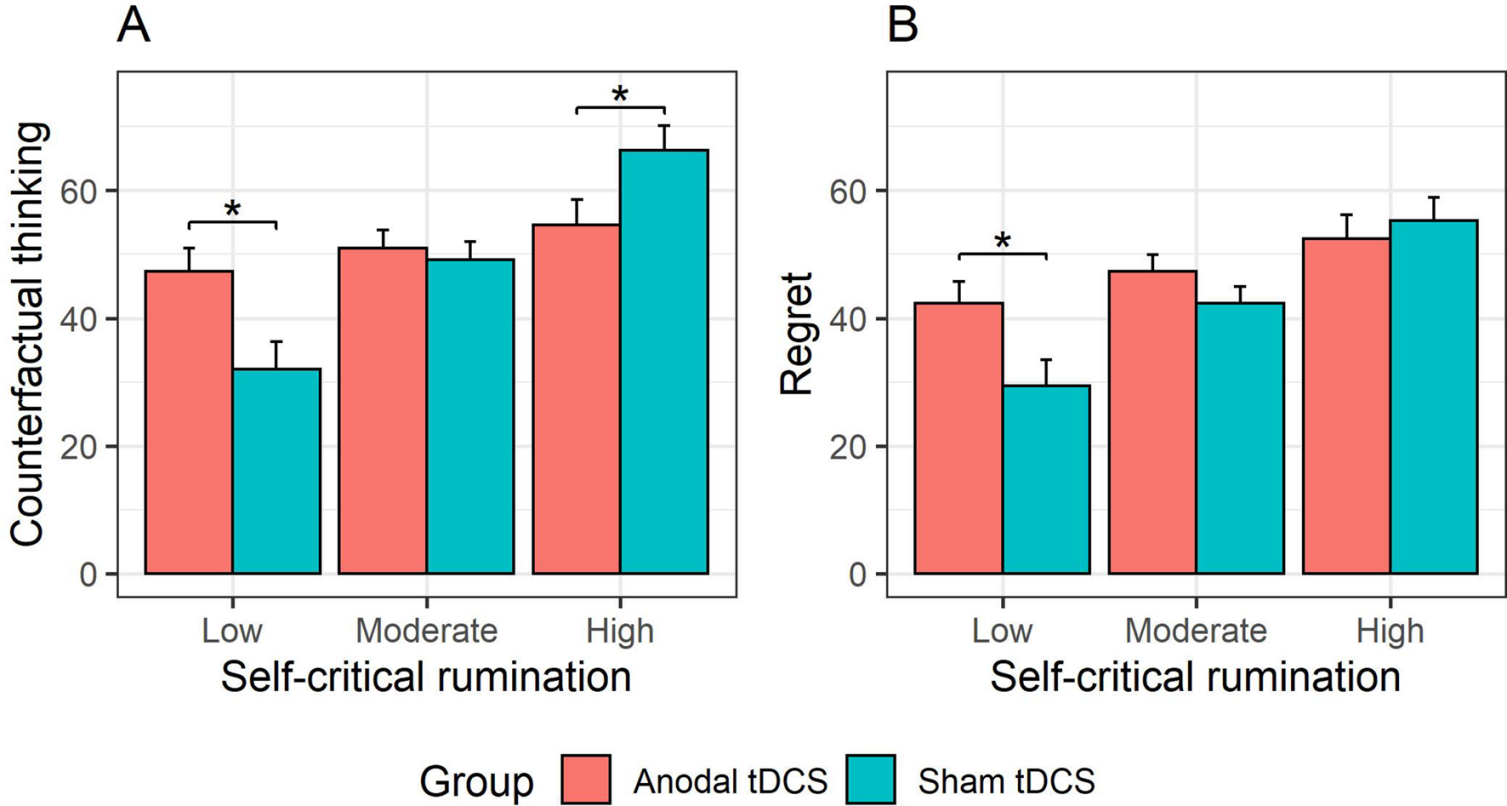
Role of level of self-critical rumination in tDCS effects on counterfactual thinking (**A**) and regret (**B**). Error bars reflect the standard error. *p < 0.05.

**Figure 4:**
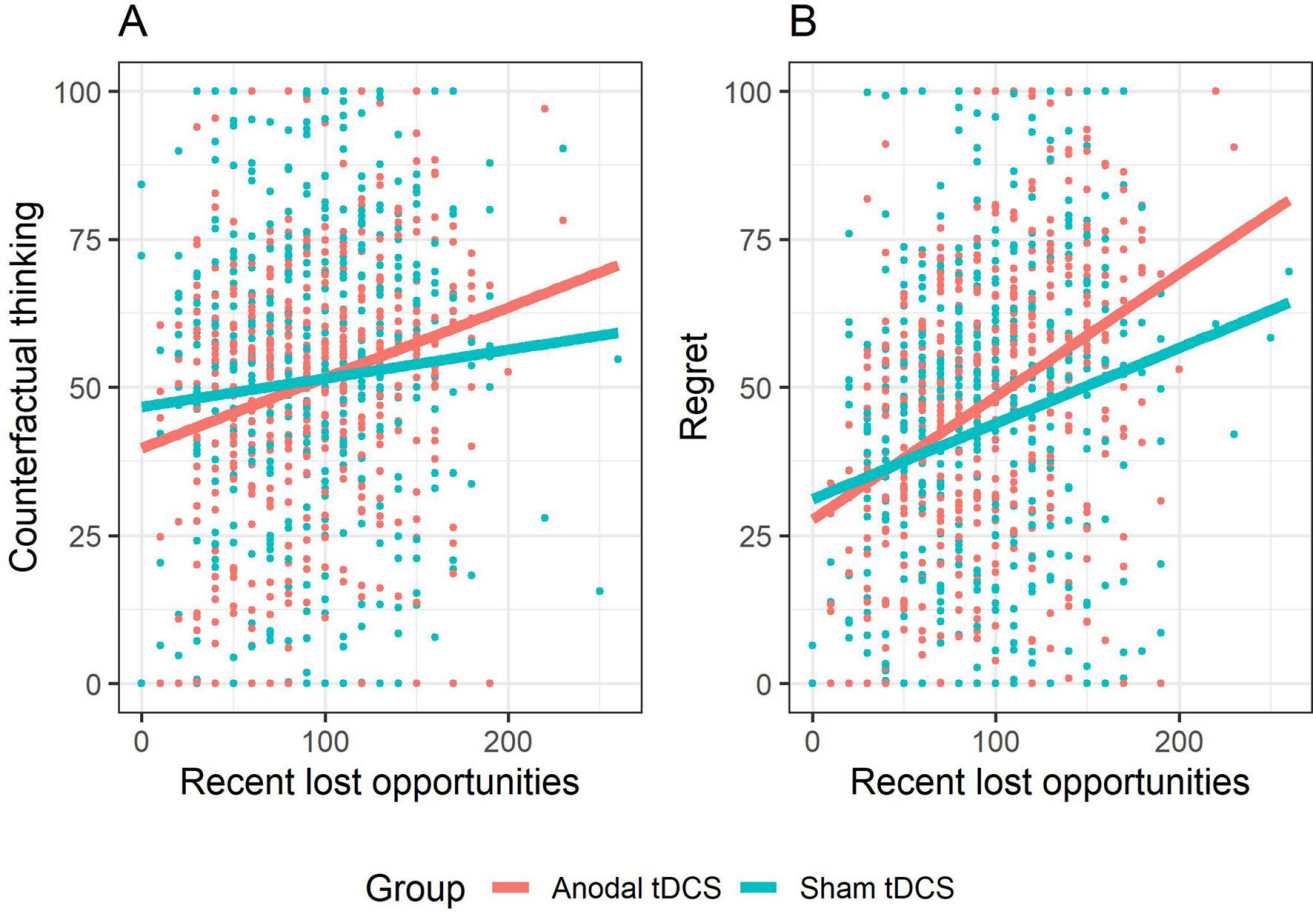
Role of recent lost opportunities in tDCS effects on counterfactual thinking.

The LMM showed that *self-critical rumination* was positively associated with counterfactual thinking, β = 10.39, *SE* = 1.99, *t* = 5.23, *p* < 0.001. Furthermore, the LMM showed a *group* × *self-critical rumination* interaction, *F*(1, 75) = 11.49, *p* = 0.001. Follow-up pairwise comparisons of the EMMs at low (*M* − 1 *SD*), moderate (M) and high (*M* + 1 *SD*) levels of self-critical rumination (Fig. 3A) showed that anodal tDCS (compared to sham) was associated with decreased CFT at a high self-critical rumination level, *b* = -11.63, *SE* = 5.56, *t* = − 2.09, *p* = 0.04, whereas at a low level, anodal tDCS was associated with increased CFT, *b* = 15.32, *SE* = 5.63, *t* = 2.72, *p* = 0.01. At a moderate level, there was no significant difference between anodal and sham tDCS, *b* = 1.84, *SE* = 3.94, *t* = 0.47, *p* = 0.64. The LMM also showed a *group* × *recent lost opportunities* interaction, *F*(1, 1898.41) = 13.82, *p* < 0.001 (Fig. 4A). Follow-up tests indicated that recent lost opportunities were positively associated with counterfactual thinking in both the sham, β = 1.35, *SE* = 0.49, *t* = 2.75, *p* = 0.01, and anodal tDCS group, β = 3.93, *SE* = 0.49, *t* = 8.02, *p* < 0.001, but this association was stronger in the anodal tDCS group, β = 2.58, *SE* = 0.69, *t* = 3.72, *p* < 0.001. All remaining tDCS effects (i.e., *group, group* × *self-critical rumination* × *recent lost opportunities*) were non-significant (all *p*s > 0.26). This model (excluding the random subject intercept) accounted for 21% of the observed variance in counterfactual thinking during the task.

#### Regret

This LMM showed that *self-critical rumination* was positively associated with regret, β = 9.01, *SE* = 1.88, *t* = 4.80, *p* < 0.001. Furthermore, it also showed a *group* × *self-critical rumination* interaction, *F*(1, 75) = 4.36, *p* = 0.04. Follow-up pairwise comparisons of the EMMs at low (*M *− 1 *SD*), moderate (M) and high (*M* + 1 *SD*) levels of self-critical rumination (Fig. 3B) showed that anodal tDCS (compared to sham) did not significantly differ on regret at high, *b* = − 2.78, *SE* = 5.56, *t* = − 0.53, *p* = 0.60, and moderate levels, *b* = 5.07, *SE* = 3.72, *t* = 1.36, *p* = 0.18, of self-critical rumination. However, at low levels, anodal tDCS was associated with increased regret, *b* = 12.91, *SE* = 5.32, *t* = 2.42, *p* = 0.02. In addition, a *group* × *recent lost opportunities* interaction was observed, *F*(1, 1903.32) = 6.51, *p* = 0.01 (Fig. 4B). Similarly, follow-up tests indicated that recent lost opportunities were positively associated with regret in both the sham, β = 5.03, *SE* = 0.62, *t* = 8.07, *p* < 0.001, and anodal tDCS group, β = 7.27, *SE* = 0.62, *t* = 11.71, *p* < 0.001, but this association was stronger in the anodal tDCS group, β = 2.24, *SE* = 0.88, *t* = 2.55, *p* = 0.01. All remaining tDCS effects (i.e., *group, group* × *self-critical rumination* × *recent lost opportunities*) were non-significant (all *p*s > 0.26). This model accounted for 17% of the observed variance in regret during the task.

### Effects of tDCS on psychophysiological reactivity to trial-based choice outcomes

#### Skin conductance responses

**Figure 5:**
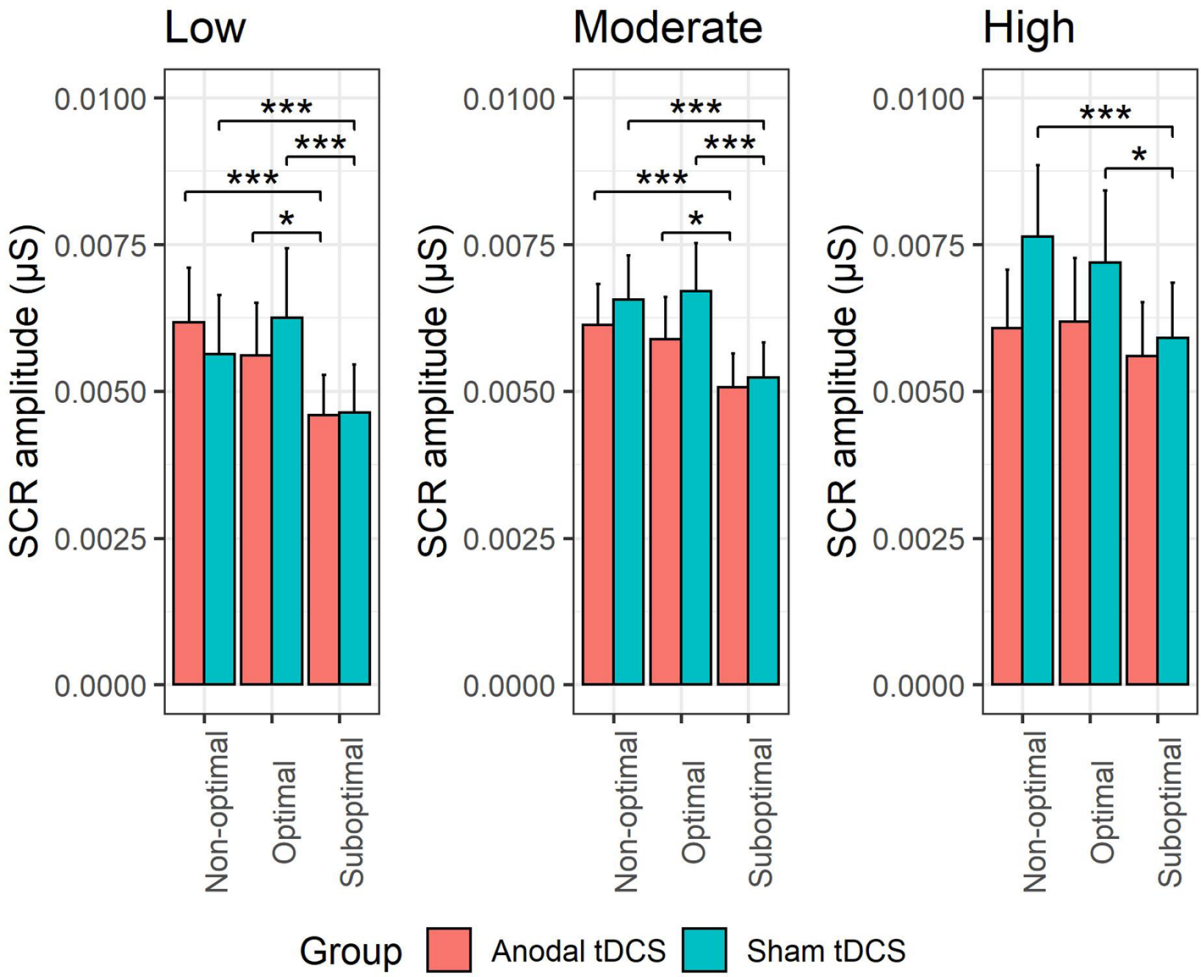
Role of level of self-critical rumination in tDCS effects on SCRs to choice outcomes. Error bars reflect the standard error. *p < 0.05; *** *p* < 0.001.

This GLMM showed a *group* × *choice outcome* × *self-critical rumination* interaction (Fig. 5), *χ*^2^(2) = 9.73, *p* = 0.01. Follow-up pairwise comparisons of the *group* × *choice outcome* interaction EMMs at low (*M *− 1 *SD*), moderate (*M*) and high (*M* + 1 *SD*) levels of self-critical rumination showed no direct significant difference between anodal and sham tDCS on any choice outcome, at any level of self-critical rumination (Supplementary Table S2; all *p*s > 0.69). Pairwise comparisons of *choice outcome* at each level of *group* and *self-critical rumination* (Supplementary Table S3) showed that SCRs were modulated by choice outcome (optimal vs suboptimal and loss vs suboptimal) in both the anodal and sham tDCS group, at low and moderate self-critical rumination levels. However, at high levels of self-critical rumination, in the anodal tDCS group, choice outcome didn’t modulate SCRs, whereas choice outcome did modulate SCRs in the sham tDCS group at this level. Specifically, in the sham tDCS group at high levels of self-critical rumination, SCRs were higher for ‘loss’ vs ‘suboptimal’ choice outcomes, *b* = 0.26, *SE* = 0.05, *t* = 5.65, *p* < 0.001, and ‘optimal’ vs ‘suboptimal’ choice outcomes, *b* = 0.20, *SE* = 0.07, *t* = 2.73, *p* = 0.04, whereas in the anodal tDCS group, this was not the case, *b* = 0.08, *SE* = 0.05, *t* = 1.78, *p* = 0.38, and, *b* = 0.10, *SE* = 0.07, *t* = 1.30, *p* = 0.58, respectively. All remaining tDCS effects (i.e., *group, group* × *self-critical rumination*, *group* × *choice outcome*) were non-significant (all *p*s > 0.39). This model (excluding the random subject intercept) accounted for 16% of the observed variance in cardiovascular reactivity.

#### Cardiovascular reactivity

**Figure 6:**
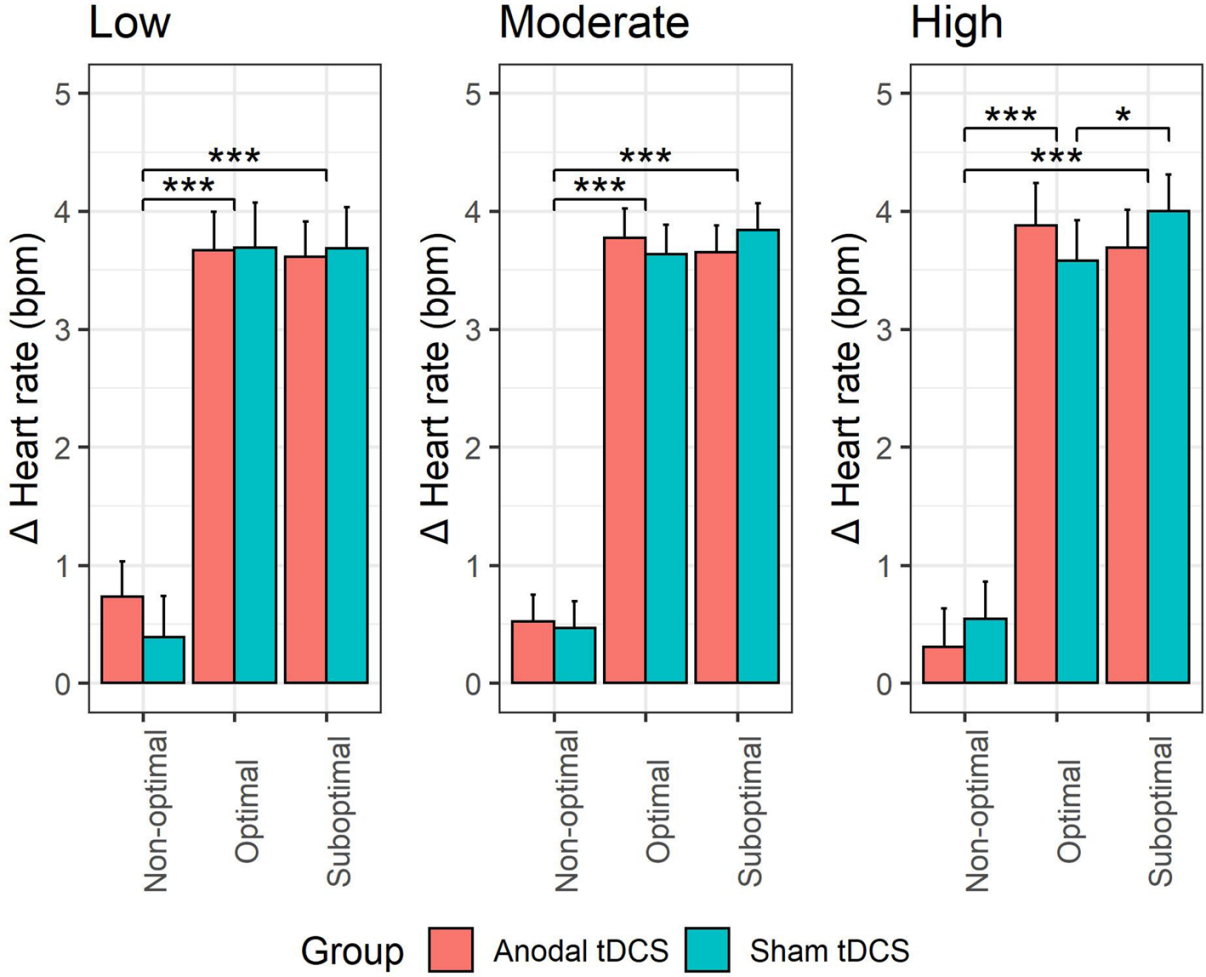
Role of level of self-critical rumination in tDCS effects on cardiovascular reactivity to choice outcomes. Error bars reflect the standard error. *p < 0.05; ***p < 0.001.

This LMM showed a *group* × *choice outcome* interaction, *F*(2, 63,863) = 3.24, *p* = 0.04. However, this interaction was driven by a higher order *group* × *choice outcome* × *self-critical rumination* interaction (Fig. 6), *F*(2, 63,866) = 3.60, *p* = 0.03. Follow-up pairwise comparisons of the *group* × *choice outcome* interaction EMMs at low (*M *− 1 *SD*), moderate (*M*) and high (*M* + 1 *SD*) levels of self-critical rumination (Supplementary Table S4) again showed no direct significant difference between anodal and sham tDCS on any choice outcome, at any level of self-critical rumination (all *p*s > 0.75). Pairwise comparisons of *choice outcome* at each level of *group* and *self-critical rumination* (Supplementary Table S5) showed that heart rate changes were modulated by choice outcome (loss vs optimal and loss vs suboptimal) across all levels of self-critical rumination and in both tDCS groups (all *p*s < 0.001). However, at high self-critical rumination levels, in the sham group, heart rate acceleration was significantly larger during ‘suboptimal’ versus ‘optimal’ choice outcomes, *b* = 0.42, *SE* = 0.17, *t* = 2.46, *p* = 0.02, and this was not the case in the anodal tDCS group at this rumination level, *b* = 0.19, *SE* = 0.18, *t* = 1.05, *p* = 0.44. Furthermore, a *group* × *time* × *self-critical rumination* interaction was present, which is presented in “Supplementary Materials”. All remaining tDCS effects (i.e., group, group × self-critical rumination, group × time, group × choice outcome × time, group × choice outcome × self-critical rumination × time) were non-significant (all ps > 0.34). This model (excluding the random subject intercept) accounted for 9% of the observed variance in SCRs.

### Construct validity of the psychophysiological measurements

To assess the construct validity of the psychophysiological measurements in their ability to capture reactivity related to counterfactual thinking and regret, a series of post-hoc analyses were conducted (see “Supplementary Materials”). These analyses showed that SCRs were positively associated with CFT, *b* = 2.12, *SE* = 0.48, *t* = 4.44, *p* < 0.001, and regret, *b* = 2.08, *SE* = 0.62, *t* = 3.35, *p* < 0.001, and the change in heart rate was negatively associated with CFT, *b* = − 1.85, *SE* = 0.39, *t* = − 4.74, *p* < 0.001, and regret, *b* = − 2.17, *SE* = 0.51, *t* = − 4.26, *p* < 0.001, thereby providing empirical support for their construct validity.

### Role of depressive symptomatology

In view of both the tight theoretical link between rumination and depression^7^, and the observed positive association between self-critical rumination and depressive symptoms, β = 4.39, *SE* = 0.78, *t* = 5.61, *p* < 0.001, a series of post-hoc analyses were conducted to verify whether the main conclusions remain when controlling for the effect of depressive symptoms (see “Supplementary Materials”). This was the case, suggesting that self-critical rumination specifically drives the observed results.

## Discussion

The goal of the present study was to investigate whether, as a function of habitual tendencies towards self-critical rumination, anodal tDCS over the left DLPFC momentarily attenuates CFT and regret, and to explore psychophysiological correlates of this hypothesized tDCS effect in response to varying choice outcomes related to goal progress (i.e., non-optimal, suboptimal, optimal). First, the finding that individuals who are prone to ruminate are also more prone to experience CFT and regret^3,4^, was replicated in the current study as evidenced by a positive association between self-critical rumination and reported CFT and regret among participants receiving sham tDCS. Furthermore, analyses showed that the physiological responses (SCR, cardiovascular reactivity) were correlated with self-reported CFT and regret, thereby establishing construct validity of the employed psychophysiological measures to capture processes related to CFT and regret.

Based on the *self-report assessments,* among individuals inclined to engage in self-critical rumination, anodal (versus sham) left DLPFC tDCS was associated with decreased CFT, but not with decreased regret. In contrast, among individuals with low rumination inclinations, anodal (versus sham) tDCS was associated with both increased regret and CFT. Among individuals with moderate rumination inclinations, anodal (versus sham) tDCS was not associated with differences in CFT or regret. Taken together, these results provide evidence that the effect of anodal tDCS over the left DLPFC on CFT and regret depends on the habitual inclination towards self-critical rumination. Furthermore, the level of self-critical rumination also plays a crucial role when investigating the effects of tDCS on the psychophysiological measurements.

Based on the *assessments of SCRs*, across all levels of self-critical rumination, sham tDCS was associated with (a) larger SCRs for non-optimal versus suboptimal outcomes, and (b) larger SCRs for optimal versus non-optimal outcomes. These findings are consistent with the notions that (a) no goal progress (i.e., non-optimal) is more emotionally arousing than making some goal progress (i.e., suboptimal), and (b) optimal goal progress is more arousing than some goal progress (i.e., suboptimal). Yet, anodal (versus sham) tDCS over the left DLPFC attenuated this SCR reactivity to the various choice outcomes (e.g., non-optimal, suboptimal, optimal), exclusively among individuals prone to self-critical rumination. Specifically, among rumination-prone individuals who received anodal tDCS, emotional arousal did not differ between suboptimal and optimal outcomes, and between suboptimal and non-optimal outcomes. This may suggest that, among self-critical rumination-inclined individuals, tDCS may have induced blunted emotional arousal to choice outcomes, as if individuals were indifferent for either relative positive or negative utility of their specific choice outcomes. Such reasoning is consistent with the above described finding that self-reported counterfactual thinking was attenuated by tDCS in this group, suggesting reduced psychological responsiveness to information of choice outcome.

Based on the *assessments of cardiovascular reactivity*, HR accelerations were significantly smaller during non-optimal outcomes compared to suboptimal or optimal outcomes, regardless of tDCS group or self-critical rumination tendencies. These findings are in line with past research showing attenuated HR acceleration or HR slowing to non-optimal outcomes^1,76^, negative feedback regarding motor performance^77^, and negative social feedback^49,50,78^, and this is thought to reflect a defensive preparatory mechanism in the face of threat or other aversive situations^43,45,51,79^. Furthermore, among individuals with high self-critical rumination tendencies who received sham tDCS, HR accelerations were larger during suboptimal versus to optimal outcomes. This was not the case among individuals with high self-critical rumination tendencies who received anodal tDCS, or among low or moderately predisposed individuals, irrespective of their tDCS group. Possibly, this enlargement of HR acceleration during suboptimal versus optimal outcomes among self-critical ruminators may reflect an increased tendency to cognitively elaborate^44,45^ on suboptimal versus optimal outcomes. Such an explanation would be consistent with the notion that ruminators are prone to counterfactual thinking and regret in situations where a decision turns out well, but discover that another decision would have led to even better outcomes^80^, as this is the case when faced with suboptimal outcomes in the task. Interestingly, enlarged HR accelerations to emotional stimuli have been observed in several vulnerable populations, such as rumination, social anxiety and post-traumatic stress disorders^81–83^. Following this reasoning, the observed HR acceleration enlargement during suboptimal versus optimal outcomes may reflect a cardiovascular marker of the proneness to experience counterfactual thinking and regret in this population. Given that this differential HR reactivity to suboptimal versus optimal outcomes was not observed during anodal tDCS among ruminators, suggests that tDCS attenuated this differential HR reactivity. Taken together, again consistent with the results of the self-reported and SCR data, these findings suggest that increased cardiovascular reactivity to suboptimal versus optimal outcomes observed exclusively in rumination-prone individuals may be a cardiovascular marker of CFT and regret-proneness in this population and that tDCS over the left DLPFC can attenuate this differential reactivity.

As stated earlier, based on the self-report assessments, among individuals with low self-critical rumination tendencies anodal tDCS was associated with an increased propensity to experience counterfactual thinking and regret. However, in contrast to self-critical rumination-prone individuals, tDCS was not associated with changes in physiological reactivity to the differential choice outcomes among these individuals. Given this absence of tDCS effects on physiological reactivity in this population, it could be argued that this self-reported increased CFT and regret propensity does not necessarily reflect a maladaptive affective/cognitive outcome, as CFT and regret are generally, in healthy populations, thought to serve adaptive functions to improve future decision making^9^. Such a reasoning would also be consistent with correlational and experimental data showing that the DLPFC is involved in both counterfactual thinking^84,85^ and the maintenance of goal-oriented intentions and behaviors^86^. Furthermore, the current results show that regardless of tendencies towards self-critical rumination, perceived recent lost opportunities was more strongly related to counterfactual thinking and regret in the anodal tDCS group. This suggests that anodal tDCS over the left DLPFC generally increased the sensitivity towards counterfactual information, consistent with the role of the DLPFC in the value encoding of hypothetical outcomes from specific actions^85,87^ and maintenance and implementation of goal-oriented behavior^86^, such as attempting to make the best decisions based on prior actions. Altogether, anodal tDCS could have generally influenced prefrontal networks involved in the value encoding of (counter)factual outcomes^85,87,88^. At the same time, depending on altered fronto-limbic connectivity associated with ruminative tendencies, anodal tDCS could have increased connectivity within this fronto-limbic network^18^, as this network is involved in down-regulation of limbic reactivity^21,89,90^, which would be in line with the observed tDCS effects on physiological reactivity to choice outcomes among ruminative-prone participants. Such a reasoning is consistent with research suggesting that interindividual differences such as preexisting activation of specific neural networks, significantly influence the outcome and efficacy of tDCS applications in mental health outcomes^91^. On a neurocognitive process level, the rumination-associated hypo-activity is thought to involve impairments in attentional processes^17,92^, such as negative attentional biases and impaired disengagement from negative information. Furthermore, prefrontal tDCS has been shown to beneficially modulate these attentional processes, in turn attenuating emotional reactivity^26,93,94^ and ruminative processes^25^. Interestingly, by using a similar task as the current paradigm, attentional deployment towards factual (i.e., points that have been achieved) versus counterfactual outcomes (i.e., points that could have been achieved) has been shown to influence CFT and regret, as measured by both self-report and functional neuro-imaging^95^. Based on this empirical data, it is possible that the effect of tDCS over the left DLPFC on CFT among high self-critical ruminators may be mediated by the modulation of these attentional processes. Given that these propositions fit well into existing theory and empirical data, the current data generates new testable hypotheses for future research potentially unraveling underlying neurocognitive mechanisms in these observed tDCS effects.

Besides several strengths, such as the ecologically valid experimental paradigm, some limitations of the current study have to be mentioned. First, because female populations tend to be more rumination-prone^55^, the sample consisted exclusively of females, but at the cost of limiting the generalizability of the results. Furthermore, past research has suggested that the stage of the menstrual cycle can influence tDCS effects^96,97^, and this was not controlled for in the current design. Given the relatively large sample size, it could be argued that these hormonal effects could be balanced out between the two groups. Second, although it could be considered as a limitation that a between-subject design was employed without measuring baseline sensitivity to the experimental paradigm, this specific design was chosen to prevent habituation and desensitization to the paradigm due to repeated exposure. Moreover, to sufficiently accommodate this limitation, the tDCS groups were matched on a wide range of potential confounding variables, thereby ensuring group comparability. Third, the experimental design would have been methodologically stronger if a double-blinded tDCS procedure^98^ would have been used. Fourth, although the current sample size was significantly larger than commonly employed sample sizes in tDCS studies^29^, power analyses indicated that there was only adequate power to detect moderate to large effect sizes, and additional post-hoc power analyses reported in the “Supplementary Materials” furthermore corroborate this notion. This warrants the need to employ even larger sample sizes in future tDCS research, as tDCS effects tend to be weak and between-subject designs also inherently involve more variance compared to within-subject designs^29,97^. Finally, although the employed tDCS protocol was motivated by both previous tDCS research on rumination^25,99^ and a priori electric field simulations, some critical reflections have to be made regarding (a) the design of the tDCS groups, (b) the electrode size, and (c) the position of the cathode electrode. First, the absence of an additional anodal tDCS group in which a brain region is targeted that is not implicated in ruminative processes, hinders clear conclusions regarding the specificity of the tDCS montage on the observed rumination-dependent tDCS effects. For instance, based on the involvement of the orbitofrontal cortex (OFC) in decision outcome valuation, in a previous study it was shown that cathodal tDCS over the OFC was associated with less intense self-reported emotions to factual and counterfactual outcomes in a gambling task^100^. In this former study, the cathode was placed over the left OFC, while the anode was placed over the right lateral occipital cortex. Second, due to the relatively large electrode size in the current study, the focality of the tDCS is reduced and the contribution of brain regions neighboring the DLPFC in the observed effects is highly likely^101–103^. Nevertheless, in view of an ongoing paradigm shift towards brain network perspectives in neuroscience^104,105^ and the importance of brain networks in affective disorders^106^, a (clinical) advantage of tDCS is its ability to modulate large-scale brain networks^107–109^, rather than brain regions in isolation, but at the cost of reduced focality. Third, in the current study the cathode was placed over the right supra-orbital area, and the current distribution of tDCS is suggested to be influenced by both the position of the anode and cathode electrode^103^. Consequently, the possibility that the observed effects were driven rather by the cathode than the anode cannot be excluded and this could potentially have been mitigated by extracephalic placement of the cathode. However, computational modelling studies suggest that extracephalic cathode montages result in a more widespread current distribution^110,111^, thereby reducing focality*.* Taken together, as a variety of parameters define the current distribution of tDCS^30,103,112^, in order to clearly elucidate the neural circuitry involved in tDCS effects, future tDCS studies would greatly benefit from the concurrent use of neuro-imaging methods such as functional magnetic resonance imaging or high density electroencephalogram, and/or the application of HD-tDCS, which features increased focality^101,113,114^.

Taken together, these findings show that in a context of self-relevant decision making, left prefrontal tDCS momentarily attenuates (1) self-reported counterfactual thinking (but not regret) and (2) differential psychophysiological reactivity to choice outcomes differing in their utility towards goal progress, specifically among individuals prone to self-critical rumination. These findings extend existing tDCS literature showing adaptive effects on emotional reactivity to various (non-self-referent) emotional stimuli^35,94,115^, by employing (self-referent) personal choice outcomes as the presented stimuli. Moreover, based on the notion that excessive counterfactual thinking may fuel regretful self-critical ruminative cognitions, thereby increasing the risk for the development of depression^3,9^, these initial results may suggest that tDCS could be of therapeutic use in attenuating counterfactual thinking, specifically among individuals who are prone to repetitively and self-critically engage with these thoughts. However, further research is warranted to systematically investigate the potential prospective effects of tDCS on the temporal dynamics between counterfactual thinking, self-critical rumination, and depressive symptomatology. Nevertheless, the current results are clinically relevant because feelings of contentment regarding one’s personal life decisions are important in mental health^1,2^, and individuals increasingly experience distress surrounding personal decision making (outcomes) in modern western societies^116^, as these societies are characterized by a large number of behavioral possibilities and typically promote individualistic self-deterministic values, associated with a high pressure on individuals to achieve goals and success^15^.

## Supplementary Information

Supplementary Information.

## Data Availability

The data and analysis script are available online (https://osf.io/3pwrb/).
