# Supplementary material for: Prefrontal tDCS attenuates counterfactual thinking in female individuals prone to self-critical rumination
Source: Sci Rep. 2021 Jun 2;11:11601. doi: 10.1038/s41598-021-90677-7 (PMC8172930; doi:10.1038/s41598-021-90677-7)
Supplement: Supplementary file 1 — Supplementary Information. [file 41598_2021_90677_MOESM1_ESM.docx]

**Prefrontal tDCS attenuates counterfactual thinking in female individuals prone to self-critical rumination**

Jens Allaert ^a, b, c^, Rudi De Raedt ^c^, Frederik M van der Veen ^d^, Chris Baeken ^a, b, c, e^, Marie-Anne Vanderhasselt ^a, b, c^

^a^ Department of Head and Skin, Ghent University, University Hospital Ghent (UZ Ghent), Ghent, Belgium

^b^ Ghent Experimental Psychiatry (GHEP) lab, Ghent University, Ghent, Belgium

^c^ Department of Experimental Clinical and Health Psychology, Ghent University, Ghent, Belgium

^d^ Department of Psychology, Education and Child Studies, Erasmus University Rotterdam, Rotterdam, the Netherlands

^e^ Department of Psychiatry, Vrije Universiteit Brussel (VUB), University Hospital UZBrussel, Brussels, Belgium

* Corresponding author:

Jens Allaert

Ghent University, Campus University Hospital (UZ Ghent)

1K12F, Corneel Heymanslaan 10, 9000 Ghent, Belgium

E-mail: [jens.allaert@ugent.be](mailto:jens.allaert@ugent.be)

Tel: +32479579086

**Supplementary Materials**

**Online Survey and Pseudo-randomized Group Allocation**

A selection of variables that potentially influence responsivity to the paradigm were measured a priori. These consisted of age, habitual tendencies towards counterfactual thinking and regret (RS^1^), behavioural inhibition and reward responsiveness (BIS/BAS^2^), symptoms of depression and anxiety (MASQ30^3^), and habitual tendencies to engage in maladaptive and adaptive emotion regulatory processes (SCRS, ERQ, CERQ^4-6^). The first half of the participant sample was randomly allocated to one of the two groups. Halfway through data collection the online survey data of planned participants were monitored by the first author. Based on this data, planned participants were allocated to one of the two groups in order to minimize group differences on these variables. The group allocation was forwarded to the experimenter, with the experimenter being unaware of the participants’ results of the online survey. Table S1 presents an overview of the descriptive statistics of these variables for both groups, and the contrasts there of. No significant differences on these variables between the anodal and sham tDCS group were observed (all *p*s > .17).

| **Table S1. Descriptive statistics and welch two sample t-test statistics** | | | | |
| --- | --- | --- | --- | --- |
|  | Sham tDCS | Anodal tDCS |  |  |
| Variable | *M* (*SD*) | *M* (*SD*) | *t* | *p* |
| Age | 20.77 (1.87) | 21.35 (1.97) | 1.34 | .18 |
| Self-critical rumination SCRS | 25.50 (6.98) | 24.10 (7.96) | -.84 | .41 |
| Regret RS | 21.85 (5.28) | 20.75 (4.80) | -.98 | .33 |
| Behavioural inhibition BIS | 23.32 (3.47) | 22.23 (3.74) | -1.36 | .18 |
| Reward responsiveness BAS | 17.73 (1.78) | 17.25 (2.19) | -1.06 | .29 |
| Drive BAS | 11.12 (2.44) | 11.72 (2.40) | 1.11 | .27 |
| Fun seeking BAS | 11.40 (2.01) | 10.75 (2.20) | -1.38 | .17 |
| General distress MASQ30 | 26.00 (8.71) | 24.02 (8.91) | -1.00 | .32 |
| Anhedonic depression MASQ30 | 28.25 (7.94) | 27.02 (8.29) | -.67 | .50 |
| Anxious arousal MASQ30 | 18.18 (6.53) | 17.18 (6.44) | -.69 | .49 |
| Cognitive reappraisal ERQ | 27.95 (4.86) | 28.98 (6.73) | .78 | .44 |
| Expressive suppression ERQ | 12.32 (4.35) | 13.25 (4.21) | .97 | .34 |
| Acceptance CERQ | 14.30 (3.31) | 14.07 (2.75) | -.33 | .74 |
| Positive reappraisal CERQ | 13.90 (3.51) | 14.05 (2.99) | .21 | .84 |
| Positive refocus CERQ | 11.80 (3.38) | 11.72 (3.36) | -.10 | .92 |
| Putting into perspective CERQ | 14.03 (3.63) | 14.03 (3.33) | .00 | .99 |
| Refocus on planning CERQ | 15.22 (2.90) | 15.10 (2.83) | -.20 | .85 |
| Rumination CERQ | 14.25 (3.24) | 13.80 (3.84) | -.57 | .57 |
| Catastrophizing CERQ | 8.18 (3.69) | 7.67 (3.13) | -.65 | .52 |
| Self-blame CERQ | 12.82 (2.76) | 12.55 (3.42) | -.40 | .69 |
| Other-blame CERQ | 8.78 (3.14) | 8.60 (2.59) | -.27 | .79 |

**Data Inspection**

Due to a technical malfunction, the self-reported data during the devil’s task was missing for one subject. Consequently, the analyses with the self-reported data were run on a subset of the total sample (N = 79).

An inspection of the averaged skin conductance responses (SCRs) and cardiovascular reactivity data per subject and per choice outcome was performed. This showed that all individuals had physiological responses differing from zero to each of the three choice outcomes, suggesting that all individuals were physiologically reactive to the experimental paradigm.

In regard to the analysis of skin conductance responses, this data often follows a skewed, non-normal distribution, where a log transformation^7^ is applied to satisfy the normality assumption in linear (mixed) models (LMs and LMMs). However, it has been shown that data transformations can have problematic implications and where applicable, generalized linear mixed models (GLMMs) can be employed without the need of transformations to satisfy a normal distribution^8^. Instead, these methods require the specification of the distribution of the dependent variable (e.g., normal, gamma, etc.) and the type of relationship (e.g., linear, log) with the independent variables. Figure 1 displays the goodness of fit of SCRs under a normal distribution, and figure 2 displays the goodness of fit under a gamma distribution. Since a gamma distribution is only compatible with data containing positive numbers (excluding zero), 20 data points containing zero (.25% of all data points) were removed to assess the fit under a gamma distribution. A visual inspection of distribution fit parameters shows that a gamma (compared to normal) distribution better fits the underlying distribution of the SCR data. This was confirmed by comparing the model fit of a LMM (AIC = -55410) versus GLMM (AIC = -65779), showing that the GLMM better fit the data.

**Figure S1. Fit of normal distribution**


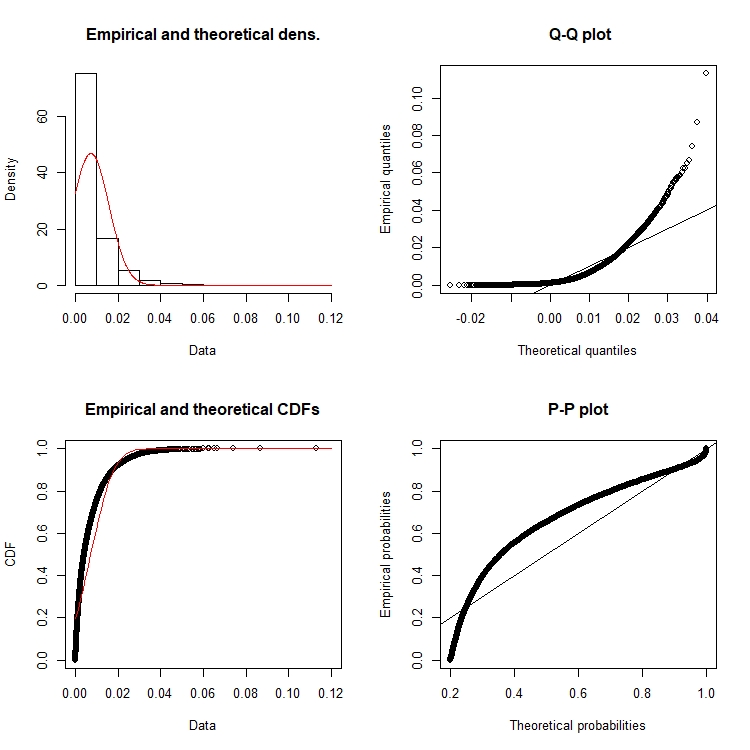


**Figure S2. Fit of gamma distribution**

**
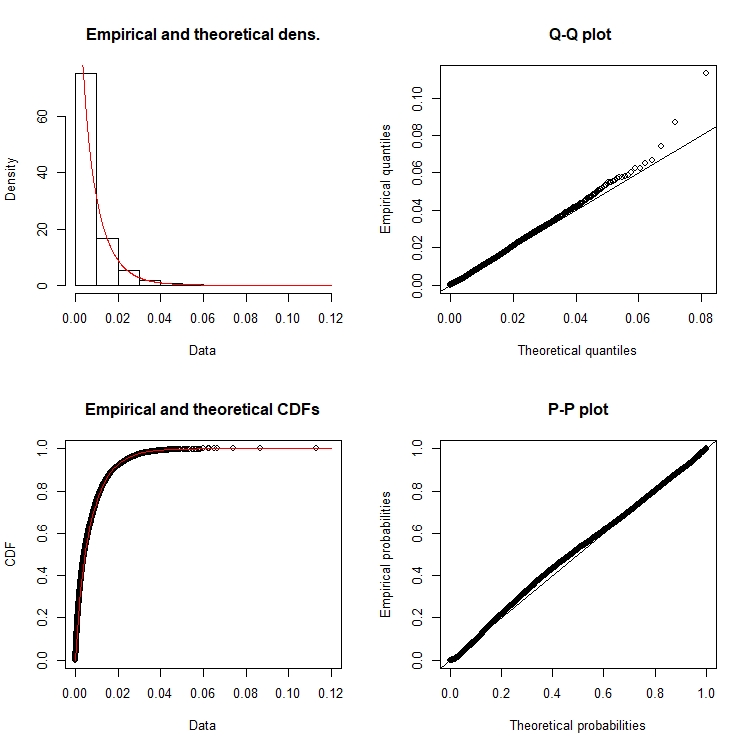
**

| **Table S2. Skin conductance responses: pairwise comparisons of tDCS groups at levels of self-critical rumination and choice outcome** | | | | |
| --- | --- | --- | --- | --- |
| Low levels of self-critical rumination (M – 1 SD) | | | | |
| Outcome | *b* | *SE* | *t* | *p* |
| Non-optimal | .09 | .23 | .40 | .87 |
| Suboptimal | -.01 | .23 | -.04 | .98 |
| Optimal | -.11 | .25 | -.44 | .87 |
| Moderate levels of self-critical rumination (M) | | | | |
| Outcome | *b* | *SE* | *t* | *p* |
| Non-optimal | -.07 | .16 | -.42 | .72 |
| Suboptimal | -.03 | .16 | -.20 | .84 |
| Optimal | -.14 | .17 | -.76 | .71 |
| High levels of self-critical rumination (M + 1 SD) | | | | |
| Outcome | *b* | *SE* | *t* | *p* |
| Non-optimal | -.23 | .23 | -.99 | .69 |
| Suboptimal | -.05 | .23 | -.24 | .90 |
| Optimal | -.15 | .24 | -.62 | .73 |

| **Table S3. Skin conductance responses: pairwise comparisons of choice outcomes at levels of self-critical rumination and tDCS group** | | | | | | | | | | |
| --- | --- | --- | --- | --- | --- | --- | --- | --- | --- | --- |
| Low levels of self-critical rumination (M – 1 SD) | | | | | | | | | | |
|  | Anodal tDCS | | | |  | Sham tDCS | | | | |
| Comparison | *b* | *SE* | *t* | *p* |  | *b* | *SE* | *t* | *p* |  |
| Non-optimal - suboptimal | .30 | .04 | 7.03 | <.001 |  | .19 | .05 | 3.93 | <.001 |  |
| Non-optimal - optimal | .10 | .07 | 1.38 | .40 |  | -.10 | .08 | -1.28 | .40 |  |
| Optimal - suboptimal | .20 | .07 | 2.84 | .02 |  | .30 | .08 | 1.71 | .001 |  |
| Moderate levels of self-critical rumination (M) | | | | | | | | | | |
|  | Anodal tDCS | | | |  | Sham tDCS | | | | |
| Comparison | *b* | *SE* | *t* | *P* |  | *b* | *SE* | *t* | *p* |  |
| Non-optimal - suboptimal | .19 | .03 | 5.89 | <.001 |  | .23 | .03 | 6.93 | <.001 |  |
| Non-optimal - optimal | .04 | .05 | .75 | .71 |  | -.02 | .05 | -.43 | .72 |  |
| Optimal - suboptimal | .15 | .05 | 2.79 | .02 |  | .25 | .05 | 4.75 | <.001 |  |
| High levels of self-critical rumination (M + 1 SD) | | | | | | | | | | |
|  | Anodal tDCS | | | |  | Sham tDCS | | | | |
| Comparison | *b* | *SE* | *t* | *p* |  | *b* | *SE* | *t* | *p* |  |
| Non-optimal - suboptimal | .08 | .05 | 1.78 | .37 |  | .26 | .04 | 5.65 | <.001 |  |
| Non-optimal - optimal | -.02 | .08 | -.23 | .90 |  | .06 | .07 | .81 | .70 |  |
| Optimal - suboptimal | .10 | .08 | 1.30 | .58 |  | .19 | .07 | 2.73 | .04 |  |

| **Table S4. Cardiovascular reactivity: pairwise comparisons of tDCS groups at levels of self-critical rumination and choice outcome** | | | | |
| --- | --- | --- | --- | --- |
| Low levels of self-critical rumination (M – 1 SD) | | | | |
| Outcome | *b* | *SE* | *t* | *p* |
| Non-optimal | .34 | .46 | .75 | .76 |
| Suboptimal | -.07 | .46 | -.15 | .97 |
| Optimal | -.03 | .50 | -.05 | .97 |
| Moderate levels of self-critical rumination (M) | | | | |
| Outcome | *b* | *SE* | *t* | *p* |
| Non-optimal | .05 | .32 | .16 | .93 |
| Suboptimal | -.19 | .32 | -.59 | .76 |
| Optimal | .14 | .35 | .39 | .87 |
| High levels of self-critical rumination (M + 1 SD) | | | | |
| Outcome | *b* | *SE* | *t* | *p* |
| Non-optimal | .24 | .45 | -.53 | .69 |
| Suboptimal | -.31 | .45 | -.68 | .68 |
| Optimal | .30 | .49 | .61 | .68 |

| **Table S5. Cardiovascular reactivity: pairwise comparisons of choice outcomes at levels of self-critical rumination and tDCS group** | | | | | | | | | |
| --- | --- | --- | --- | --- | --- | --- | --- | --- | --- |
| Low levels of self-critical rumination (M – 1 SD) | | | | | | | | | |
|  | Anodal tDCS | | | |  | Sham tDCS | | | |
| Comparison | *b* | *SE* | *t* | *p* |  | *b* | *SE* | *t* | *p* |
| Non-optimal - suboptimal | -2.88 | .10 | -28.96 | <.001 |  | -3.29 | .11 | -28.07 | <.001 |
| Non-optimal - optimal | -2.93 | .17 | -17.56 | <.001 |  | -3.30 | .19 | -17.26 | <.001 |
| Optimal - suboptimal | .05 | .17 | .31 | .97 |  | .01 | .19 | .04 | .97 |
| Moderate levels of self-critical rumination (M) | | | | | | | | | |
|  | Anodal tDCS | | | |  | Sham tDCS | | | |
| Comparison | *b* | *SE* | *t* | *P* |  | *b* | *SE* | *t* | *p* |
| Non-optimal - suboptimal | -3.13 | .08 | -41.14 | <.001 |  | -3.37 | .08 | -44.26 | <.001 |
| Non-optimal - optimal | -3.25 | .13 | -25.73 | <.001 |  | -3.17 | .12 | -25.70 | <.001 |
| Optimal - suboptimal | .12 | .13 | .96 | .51 |  | -.20 | .12 | -1.66 | .16 |
| High levels of self-critical rumination (M + 1 SD) | | | | | | | | | |
|  | Anodal tDCS | | | |  | Sham tDCS | | | |
| Comparison | *b* | *SE* | *t* | *p* |  | *b* | *SE* | *t* | *p* |
| Non-optimal - suboptimal | -3.38 | .11 | -30.75 | <.001 |  | -3.45 | .11 | -32.64 | <.001 |
| Non-optimal - optimal | -3.57 | .18 | -19.97 | <.001 |  | -3.04 | .17 | -17.77 | <.001 |
| Optimal - suboptimal | .19 | .18 | 1.05 | .44 |  | -.41 | .17 | -2.46 | .02 |

**Additional tDCS effects on cardiovascular reactivity**

A *group* × *time* × *self-critical rumination* interaction (Fig. S5) , *F*(7, 63828) = 3.08, *p* = .003, was observed. Follow-up pairwise comparisons of the *group* × *time* interaction EMMs at high (*M* + 1 *SD*) levels of self-critical rumination (supplementary table S6 & S7) showed that sham tDCS was associated with significant differences in heart rate changes between a) T2 and T7, *b* = .55, *SE* = .25, *t* = 2.23, *p* = .03, b) T3 and T5, *b* = .93, *SE* = .25, *t* = 3.78, *p* < .001, and c) T7 and T8, *b* = .69, *SE* = .25, *t* = 2.80, *p* = .01, whereas these differences were not present in the anodal tDCS group (all *p*s > .05). Pairwise comparisons at low (*M* – 1 *SD*) levels of self-critical rumination showed that anodal tDCS was associated with significant differences in heart rate changes between a) T2 and T8, *b* = 1.06, *SE* = .24, *t* = 4.39, *p* < .001, b) T3 and T5, *b* = 1.14, *SE* = .24, *t* = 4.74, *p* < .001, T4 and T5, *b* = .97, *SE* = .24, *t* = 4.02, *p* < .001, and T7 and T8, *b* = .60, *SE* = .24, *t* = 2.49, *p* = .02, whereas these differences were not present in the sham tDCS group (all *p*s > .20). However, in the sham group, there was a significant difference between T2 and T6, *b* = -1.01, *SE* = .28, *t* = 3.66, *p* < .001, which was not present in the anodal group, *b* = -.04, *SE* = .24, *t* = .18, *p* = .86. At moderate levels of self-critical rumination, pairwise comparisons showed that only in the sham tDCS group there was a significant difference in heart rate changes between T2 and T6, *b* = -.62, *SE* = .18, *t* = 3.47, *p* < .001, whereas only in the anodal tDCS group there was a significant difference between T2 and T7, *b* = -.26, *SE* = .18, *t* = -1.40, *p* = .17.

**Figure S5. Effect of time on cardiovascular reactivity at levels of self-critical rumination in the sham versus anodal tDCS group**

**
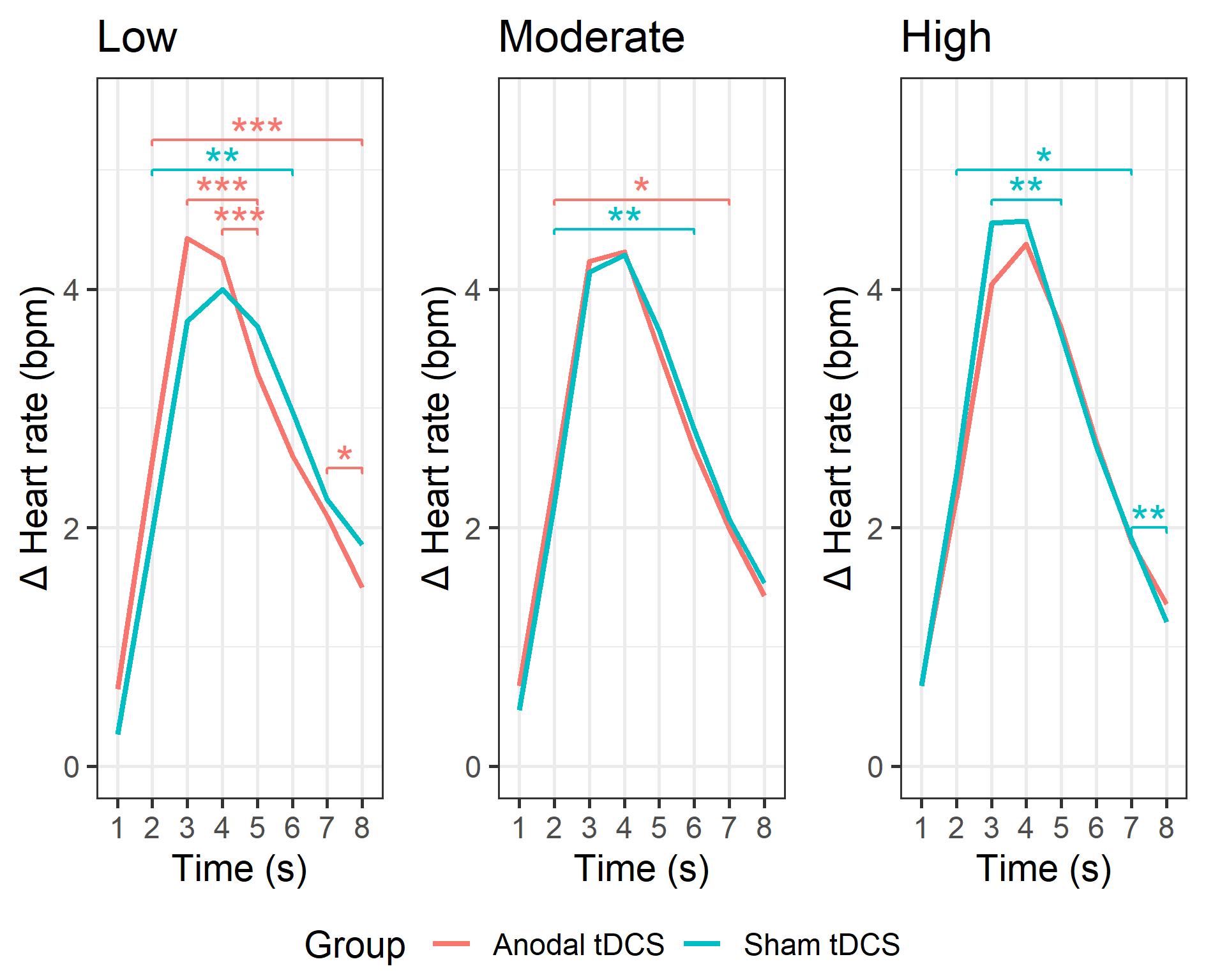
**

| **Table S6. Cardiovascular reactivity: pairwise comparisons of tDCS groups at levels of self-critical rumination and time** | | | | |
| --- | --- | --- | --- | --- |
| Low levels of self-critical rumination (M – 1 SD) | | | | |
| Time | *b* | *SE* | *t* | *p* |
| 1 | .38 | .52 | .74 | .46 |
| 2 | .60 | .52 | 1.16 | .25 |
| 3 | .70 | .52 | 1.35 | .18 |
| 4 | .26 | .52 | .50 | .62 |
| 5 | -.40 | .52 | -.77 | .43 |
| 6 | -.37 | .52 | -.72 | .47 |
| 7 | -.14 | .52 | -.27 | .78 |
| 8 | -.36 | .52 | -.69 | .49 |
| Moderate levels of self-critical rumination (M) | | | | |
| Time | *b* | *SE* | *t* | *p* |
| 1 | .20 | .36 | .56 | .57 |
| 2 | .20 | .36 | .55 | .58 |
| 3 | .09 | .36 | .26 | .80 |
| 4 | .03 | .36 | .09 | .93 |
| 5 | -.17 | .36 | -.48 | .63 |
| 6 | -.17 | .36 | -.46 | .65 |
| 7 | -.08 | .36 | -.23 | .82 |
| 8 | -.10 | .36 | -.29 | .77 |
| High levels of self-critical rumination (M + 1 SD) | | | | |
| Time | *b* | *SE* | *t* | *p* |
| 1 | .03 | .51 | .05 | .96 |
| 2 | -.20 | .51 | -.40 | .69 |
| 3 | -.51 | .51 | -1.01 | .31 |
| 4 | -.20 | .51 | -.39 | .70 |
| 5 | .06 | .51 | .11 | .91 |
| 6 | .04 | .51 | .08 | .93 |
| 7 | -.03 | .51 | -.05 | .96 |
| 8 | .15 | .51 | .29 | .77 |

| **Table S7. Cardiovascular reactivity: pairwise comparisons of time at levels of self-critical rumination and tDCS group** | | | | | | | | | |
| --- | --- | --- | --- | --- | --- | --- | --- | --- | --- |
| Low levels of self-critical rumination (M – 1 SD) | | | | | | | | | |
|  | Anodal tDCS | | | |  | Sham tDCS | | | |
| Comparison | *b* | *SE* | *t* | *p* |  | *b* | *SE* | *t* | *p* |
| 1 – 2 | -1.91 | .24 | -7.95 | <.001 |  | -1.69 | .28 | -6.11 | <.001 |
| 1 – 3 | -3.78 | .24 | -15.73 | <.001 |  | -3.46 | .28 | -12.52 | <.001 |
| 1 – 4 | -3.61 | .24 | -15.01 | <.001 |  | -3.73 | .28 | -13.48 | <.001 |
| 1 – 5 | -2.64 | .24 | -10.99 | <.001 |  | -3.42 | .28 | -12.36 | <.001 |
| 1 – 6 | -1.65 | .24 | -8.13 | <.001 |  | -2.70 | .28 | -9.77 | <.001 |
| 1 – 7 | -1.45 | .24 | -6.04 | <.001 |  | -1.97 | .28 | -7.13 | <.001 |
| 1 – 8 | -.85 | .24 | -3.55 | <.001 |  | -1.59 | .28 | -5.75 | <.001 |
| 2 – 3 | -1.87 | .24 | -7.79 | <.001 |  | -1.77 | .28 | -6.41 | <.001 |
| 2 – 4 | -1.70 | .24 | -7.07 | <.001 |  | -2.04 | .28 | -7.37 | <.001 |
| 2 – 5 | -.73 | .24 | -3.05 | .003 |  | -1.73 | .28 | -6.25 | <.001 |
| 2 – 6 | -.04 | .24 | -1.18 | .86 |  | -1.01 | .28 | -3.66 | <.001 |
| 2 – 7 | .46 | .24 | 1.91 | .06 |  | -.28 | .28 | -1.01 | .35 |
| 2 – 8 | 1.06 | .24 | 4.39 | <.001 |  | .10 | .28 | .36 | .74 |
| 3 – 4 | .17 | .24 | .72 | .49 |  | -.26 | .28 | -.96 | .36 |
| 3 – 5 | 1.14 | .24 | 4.74 | <.001 |  | .04 | .28 | .16 | .88 |
| 3 – 6 | 1.83 | .24 | 7.61 | <.001 |  | .76 | .28 | 2.75 | .01 |
| 3 – 7 | 2.33 | .24 | 9.69 | <.001 |  | 1.49 | .28 | 5.36 | <.001 |
| 3 – 8 | 2.93 | .24 | 12.18 | <.001 |  | 1.87 | .28 | 6.77 | <.001 |
| 4 – 5 | .67 | .24 | 4.02 | <.001 |  | .30 | .28 | 1.11 | .31 |
| 4 – 6 | 1.66 | .24 | 6.89 | <.001 |  | 1.03 | .28 | 3.71 | <.001 |
| 4 – 7 | 2.16 | .24 | 8.97 | <.001 |  | 1.76 | .28 | 6.35 | <.001 |
| 4 – 8 | 2.76 | .24 | 11.46 | <.001 |  | 2.14 | .28 | 7.73 | <.001 |
| 5 – 6 | .69 | .24 | 2.87 | .01 |  | .72 | .28 | 2.59 | .01 |
| 5 – 7 | 1.19 | .24 | 4.95 | <.001 |  | 1.45 | .28 | 5.24 | <.001 |
| 5 – 8 | 1.79 | .24 | 7.44 | <.001 |  | 1.83 | .28 | 6.61 | <.001 |
| 6 – 7 | .50 | .24 | 2.09 | .04 |  | .73 | .28 | 2.65 | .01 |
| 6 – 8 | 1.10 | .24 | 4.57 | <.001 |  | 1.11 | .28 | 4.02 | <.001 |
| 7 – 8 | .60 | .24 | 2.49 | .02 |  | .38 | .28 | 1.38 | .21 |
| Moderate levels of self-critical rumination (M) | | | | | | | | | |
|  | Anodal tDCS | | | |  | Sham tDCS | | | |
| Comparison | *b* | *SE* | *t* | *p* |  | *b* | *SE* | *t* | *p* |
| 1 – 2 | -1.73 | .18 | -9.48 | <.001 |  | -1.74 | .18 | -9.73 | <.001 |
| 1 – 3 | -3.56 | .18 | -19.49 | <.001 |  | -3.67 | .18 | -20.58 | <.001 |
| 1 – 4 | -3.64 | .18 | -19.94 | <.001 |  | -3.81 | .18 | -21.38 | <.001 |
| 1 – 5 | -2.81 | .18 | -15.38 | <.001 |  | -3.18 | .18 | -17.84 | <.001 |
| 1 – 6 | -1.99 | .18 | -10.88 | <.001 |  | -2.36 | .18 | -13.20 | <.001 |
| 1 – 7 | -1.32 | .18 | -7.20 | <.001 |  | -1.60 | .18 | -8.97 | <.001 |
| 1 – 8 | -.76 | .18 | -4.15 | <.001 |  | -1.06 | .18 | -5.97 | <.001 |
| 2 – 3 | -1.83 | .18 | -10.01 | <.001 |  | -1.96 | .18 | -10.85 | <.001 |
| 2 – 4 | -1.91 | .18 | -10.46 | <.001 |  | -2.08 | .18 | -11.65 | <.001 |
| 2 – 5 | -1.08 | .18 | -5.90 | <.001 |  | -1.45 | .18 | -8.11 | <.001 |
| 2 – 6 | -.26 | .18 | -1.40 | .17 |  | -.62 | .18 | -3.47 | <.001 |
| 2 – 7 | .42 | .18 | 2.28 | .02 |  | .14 | .18 | .76 | .45 |
| 2 – 8 | .97 | .18 | 5.33 | <.001 |  | .67 | .18 | 3.76 | <.001 |
| 3 – 4 | -.08 | .18 | -.44 | .65 |  | -.14 | .18 | -.80 | .44 |
| 3 – 5 | .75 | .18 | 4.11 | <.001 |  | .49 | .18 | 2.74 | .01 |
| 3 – 6 | 1.57 | .18 | 8.61 | <.001 |  | 1.31 | .18 | 7.38 | <.001 |
| 3 – 7 | 2.25 | .18 | 12.29 | <.001 |  | 2.07 | .18 | 11.60 | <.001 |
| 3 – 8 | 2.80 | .18 | 15.35 | <.001 |  | 2.61 | .18 | 14.61 | <.001 |
| 4 – 5 | .83 | .18 | 4.56 | <.001 |  | .63 | .18 | 3.54 | <.001 |
| 4 – 6 | 1.66 | .18 | 9.06 | <.001 |  | 1.46 | .18 | 8.18 | <.001 |
| 4 – 7 | 2.33 | .18 | 12.73 | <.001 |  | 2.21 | .18 | 12.40 | <.001 |
| 4 – 8 | 2.89 | .18 | 15.79 | <.001 |  | 2.75 | .18 | 15.41 | <.001 |
| 5 – 6 | .82 | .18 | 4.50 | <.001 |  | .83 | .18 | 4.64 | <.001 |
| 5 – 7 | 1.49 | .18 | 8.18 | <.001 |  | 1.58 | .18 | 8.87 | <.001 |
| 5 – 8 | 2.05 | .18 | 11.23 | <.001 |  | 2.12 | .18 | 11.87 | <.001 |
| 6 – 7 | .67 | .18 | 3.68 | <.001 |  | .75 | .18 | 4.22 | <.001 |
| 6 – 8 | 1.23 | .18 | 6.73 | <.001 |  | 1.29 | .18 | 7.23 | <.001 |
| 7 – 8 | .56 | .18 | 3.06 | .003 |  | .54 | .18 | 3.01 | .003 |
| High levels of self-critical rumination (M + 1 SD) | | | | | | | | | |
|  | Anodal tDCS | | | |  | Sham tDCS | | | |
| Comparison | *b* | *SE* | *t* | *p* |  | *b* | *SE* | *t* | *p* |
| 1 – 2 | -1.56 | .26 | -5.97 | <.001 |  | -1.78 | .25 | -7.21 | <.001 |
| 1 – 3 | -3.34 | .26 | -12.84 | <.001 |  | -3.88 | .25 | -15.71 | <.001 |
| 1 – 4 | -3.67 | .26 | -14.13 | <.001 |  | -3.90 | .25 | -15.79 | <.001 |
| 1 – 5 | -2.98 | .26 | -11.44 | <.001 |  | -2.95 | .25 | -11.93 | <.001 |
| 1 – 6 | .2.02 | .26 | -7.77 | <.001 |  | -2.01 | .25 | -8.12 | <.001 |
| 1 – 7 | -1.18 | .26 | -4.54 | <.001 |  | -1.23 | .25 | -4.99 | <.001 |
| 1 – 8 | -.66 | .26 | -2.54 | .01 |  | -.54 | .25 | -2.18 | .03 |
| 2 – 3 | -1.79 | .26 | -6.87 | <.001 |  | -2.10 | .25 | -8.50 | <.001 |
| 2 – 4 | -2.12 | .26 | -8.16 | <.001 |  | -2.12 | .25 | -8.58 | <.001 |
| 2 – 5 | -1.42 | .26 | -5.47 | <.001 |  | -1.17 | .25 | -4.72 | <.001 |
| 2 – 6 | -.47 | .26 | -1.80 | .08 |  | -.23 | .25 | -.91 | .37 |
| 2 – 7 | .37 | .26 | 1.44 | .16 |  | .55 | .25 | 2.23 | .03 |
| 2 – 8 | .89 | .26 | 3.43 | <.001 |  | 1.24 | .25 | 5.03 | <.001 |
| 3 – 4 | -.34 | .26 | -1.29 | .20 |  | -.02 | .25 | -.08 | .93 |
| 3 – 5 | .37 | .26 | 1.40 | .17 |  | .93 | .25 | 3.78 | <.001 |
| 3 – 6 | 1.32 | .26 | 5.07 | <.001 |  | 1.87 | .25 | 7.59 | <.001 |
| 3 – 7 | 2.16 | .26 | 8.30 | <.001 |  | 2.65 | .25 | 10.73 | <.001 |
| 3 – 8 | 2.68 | .26 | 10.30 | <.001 |  | 3.34 | .25 | 13.53 | <.001 |
| 4 – 5 | .70 | .26 | 2.69 | .01 |  | .95 | .25 | 3.86 | <.001 |
| 4 – 6 | 1.66 | .26 | 6.36 | <.001 |  | 1.89 | .25 | 7.67 | <.001 |
| 4 – 7 | 2.50 | .26 | 9.59 | <.001 |  | 2.67 | .25 | 10.81 | <.001 |
| 4 – 8 | 3.01 | .26 | 11.58 | <.001 |  | 3.36 | .25 | 13.61 | <.001 |
| 5 – 6 | .96 | .26 | 3.67 | <.001 |  | .94 | .25 | 3.91 | <.001 |
| 5 – 7 | 1.80 | .26 | 6.90 | <.001 |  | 1.71 | .25 | 6.95 | <.001 |
| 5 – 8 | 2.32 | .26 | 8.89 | <.001 |  | 2.41 | .25 | 9.75 | <.001 |
| 6 – 7 | .84 | .26 | 3.23 | .002 |  | .77 | .25 | 3.14 | .002 |
| 6 – 8 | 1.36 | .26 | 5.23 | <.001 |  | 1.47 | .25 | 5.94 | <.001 |
| 7 – 8 | .52 | .26 | 1.99 | .05 |  | .69 | .25 | 2.80 | .01 |

**Effect of tDCS on Reported Counterfactual Thinking and Regret Post-task**

**Counterfactual thinking.** A linear model featuring reported post-task counterfactual thinking as dependent variable, *group* (sham tDCS, anodal tDCS) as fixed factor, and *self-critical rumination* as continuous predictor showed a significant *group* × *self-critical rumination* interaction, *F*(1, 71) = 6.35, *p* = .01. Follow-up pairwise comparisons of the EMMs at low (M -1 SD), moderate (M) and high (M + 1 SD) levels of self-critical rumination (see figure 3A) showed that anodal tDCS (compared to sham) did not significantly differ on counterfactual thinking at high, *b* = -9.10, *SE* = 7.16, *t* = -1.27, *p* = .21, and moderate levels, *b* = 3.89, *SE* = 5.05, *t* = .77, *p* = .44, of self-critical rumination. However, at low levels, anodal tDCS was associated with increased counterfactual thinking, *b* = 16.89, *SE* = 7.27, *t* = 2.32, *p* = .02 All remaining tDCS effects (i.e., *group, group* × *total lost opportunities*, *group* × *self-critical rumination* × *total lost opportunities*) were non-significant (all *p*s > .41). This model accounted for 22 % of the observed variance in counterfactual thinking post-task.

**Regret.** A linear model featuring reported post-task regret as dependent variable, *group* (sham tDCS, anodal tDCS) as fixed factor, and *self-critical rumination* as continuous predictor showed a significant *group* × *self-critical rumination* interaction, *F*(1, 71) = 6.63, *p* = .01. Similarly, follow-up pairwise comparisons of the EMMs at low (M -1 SD), moderate (M) and high (M + 1 SD) levels of self-critical rumination (see figure 3B) showed that anodal tDCS (compared to sham) did not significantly differ on regret at high, *b* = -7.23, *SE* = 8.09, *t* = -.89, *p* = .38, and moderate levels, *b* = 7.79, *SE* = 5.70, *t* = 1.37, *p* = .18, of self-critical rumination. However, at low levels, anodal tDCS was associated with increased counterfactual thinking, *b* = 22.81, *SE* = 8.22, *t* = 2.78, *p* = .01 All remaining tDCS effects (i.e., *group, group* × *total lost opportunities*, *group* × *self-critical rumination* × *total lost opportunities*) were non-significant (all *p*s > .18). This model accounted for 22 % of the observed variance in counterfactual thinking post-task.

It is important to note that these analyses feature substantially lower statistical power compared to the main analyses, as the dependent variable is only based on one measurement per subject while the main analyses are based on repeated measurements per subject.

**Figure S3. Role of self-critical rumination in tDCS effects on counterfactual thinking and regret post-task**


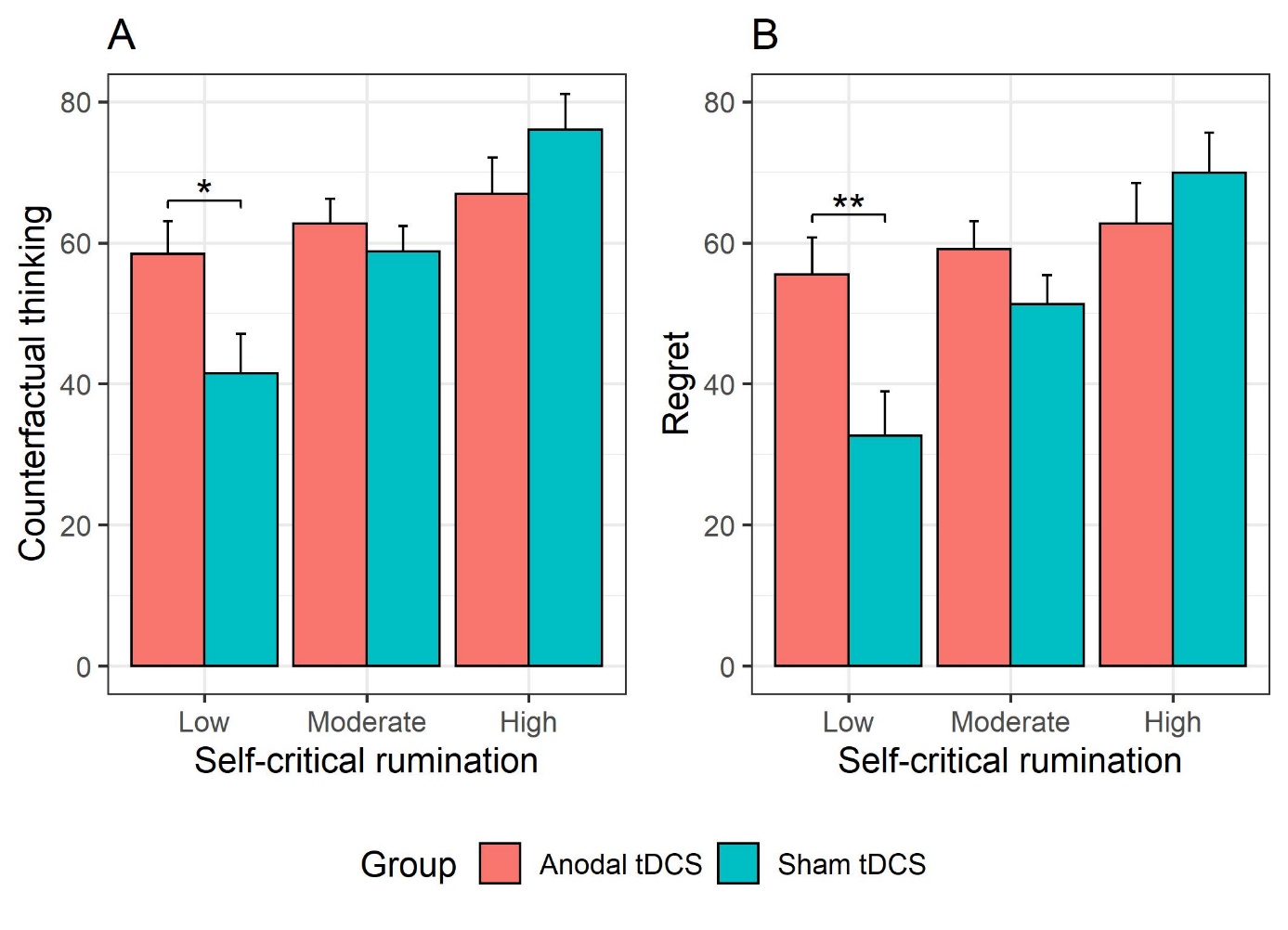


## Construct Validity of the Psychophysiological Measurements

To assess the construct validity of the psychophysiological measurements in their ability to capture reactivity related to counterfactual thinking and regret, a series of post-hoc analyses were conducted to test the linear relationships between the psychophysiological measurements (SCRs and heart rate changes) and the self-report measures (e.g., counterfactual thinking and regret). Given the difference in measurement frequency (e.g., on a trial-basis and every 4 trials) between the self-report and psychophysiological measurement types, the psychophysiological measurements of every 4 trials were averaged. This resulted in an averaged dataset that has an equal measurement frequency between the self-report and psychophysiological measurement types. For each psychological measurement type (SCRs and heart rate changes), 2 LMMs were fitted, with counterfactual thinking and regret as dependent variables, respectively, *averaged psychophysiological reactivity* as continuous predictor, and *subject* as random intercept. For SCRs, the LMMs showed a positive association between SCRs and both counterfactual thinking (Figure S4A), *b* = 2.12, *SE* = .48, *t* = 4.44, *p* < .001, and regret (Figure 3B), *b* = 2.08, *SE* = .62, *t* = 3.35, *p* < .001. For changes in heart rate, the LMMs showed a negative association between changes in heart rate and both counterfactual thinking (Figure S4C), *b* = -1.85, *SE* = .39, *t* = -4.74, *p* < .001, and regret (Figure 4D), *b* = -2.17, *SE* = .51, *t* = -4.26, *p* < .001.

**Figure S4. Association between self-report and psychophysiological measurements**


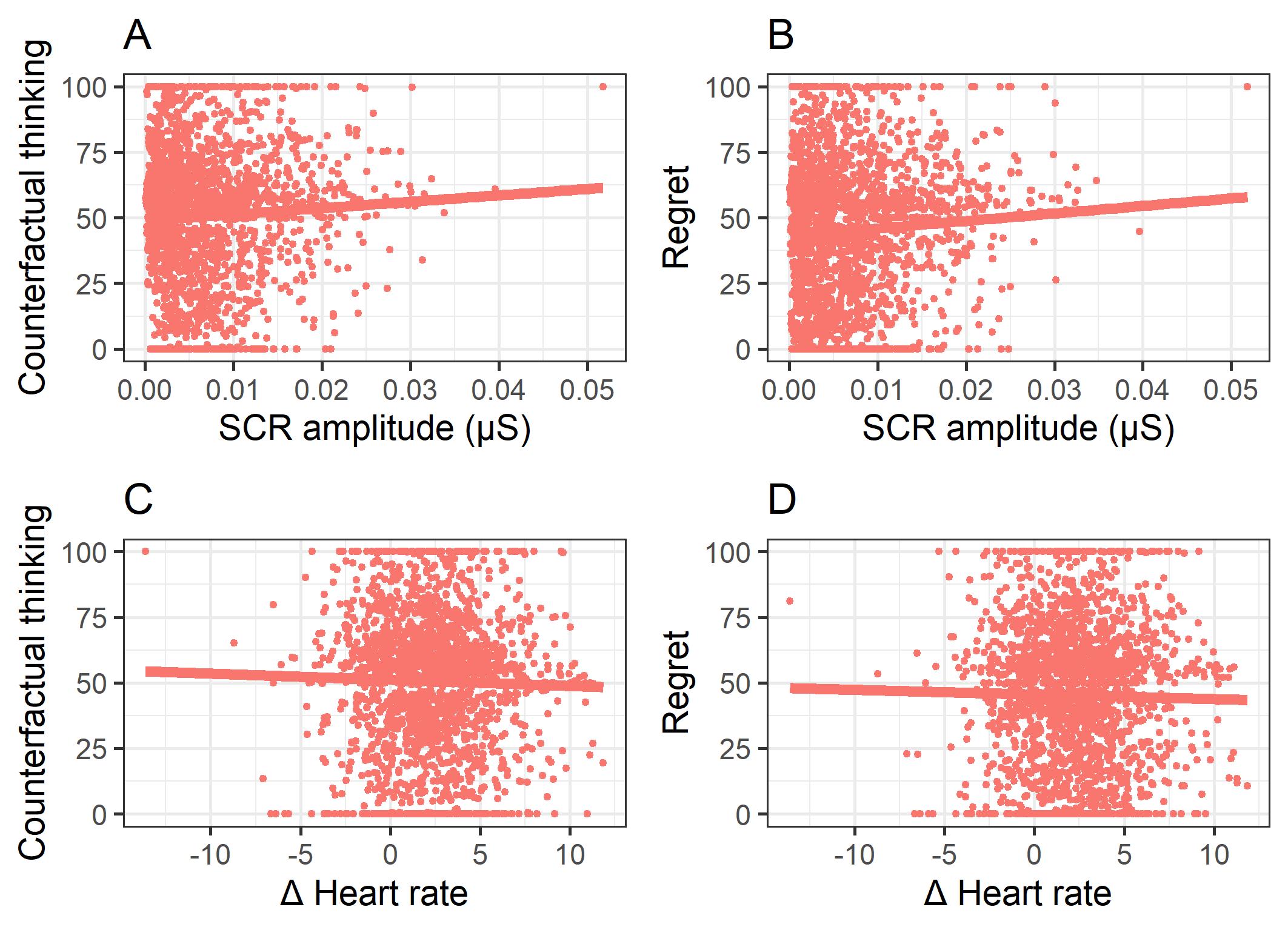


**Post-hoc Analyses with Depressive Symptoms as Control Variable**

To verify that the main observed results were independent of depressive symptoms, all main statistical models (e.g., self-reported CFT, self-reported regret, skin conductance responses and cardiovascular reactivity) were refitted with the addition of *depressive symptoms* as control variable. These analyses showed that (Table S8-S11), when controlling for depressive symptoms, all the statistical conclusions inferred from the main analyses remained the same, suggesting that depressive symptoms did not influence the observed results.

| **Table S8. Self-reported counterfactual thinking** | | | | | | |
| --- | --- | --- | --- | --- | --- | --- |
| Variable | *Sum of Squares* | *Mean Square* | *df_Num_* | *df_Den_* | *F* | *p* |
| Group | 53.0 | 53.0 | 1 | 74.00 | .24 | .62 |
| Self-critical rumination | 3805.9 | 3805.9 | 1 | 74.00 | 16.99 | <.001 |
| Recent lost opportunities | 12954.0 | 12954.0 | 1 | 1898.21 | 57.82 | <.001 |
| Anhedonic depression | 68.4 | 68.4 | 1 | 74.02 | .30 | .58 |
| Group × Self-critical rumination | 2576.3 | 2576.3 | 1 | 74.00 | 11.50 | .001 |
| Group × Recent lost opportunities | 3092.6 | 3092.6 | 1 | 1898.25 | 13.80 | <.001 |
| Self-critical rumination × Recent lost opportunities | 289.1 | 289.1 | 1 | 1898.17 | 1.29 | .26 |
| Group × Self-critical rumination × Recent lost opportunities | 183.0 | 183.0 | 1 | 1897.69 | .82 | .37 |
| *Notes*. *df_Num_* indicates degrees of freedom numerator; *df_Den_* indicates degrees of freedom denominator. | | | | | | |

| **Table S9. Self-reported regret** | | | | | | |
| --- | --- | --- | --- | --- | --- | --- |
| Variable | *Sum of Squares* | *Mean Square* | *df_Num_* | *df_Den_* | *F* | *p* |
| Group | 752 | 752 | 1 | 73.99 | 2.09 | .15 |
| Self-critical rumination | 3672 | 3672 | 1 | 74.01 | 10.20 | .002 |
| Recent lost opportunities | 70233 | 70233 | 1 | 1903.36 | 195.13 | <.001 |
| Anhedonic depression | 1115 | 1115 | 1 | 74.03 | 3.10 | .08 |
| Group × Self-critical rumination | 1703 | 1703 | 1 | 74.00 | 4.73 | .03 |
| Group × Recent lost opportunities | 2323 | 2323 | 1 | 1903.44 | 6.45 | .01 |
| Self-critical rumination × Recent lost opportunities | 1976 | 1976 | 1 | 1903.11 | 5.49 | .02 |
| Group × Self-critical rumination × Recent lost opportunities | 112 | 112 | 1 | 1902.44 | .31 | .58 |
| *Notes*. *df_Num_* indicates degrees of freedom numerator; *df_Den_* indicates degrees of freedom denominator. | | | | | | |

| **Table S10. Skin conductance responses** | | | |
| --- | --- | --- | --- |
| Variable | *χ*^2^ | *df* | *p* |
| Group | .22 | 1 | .64 |
| Choice outcome | 89.26 | 2 | <.001 |
| Self-critical rumination | .43 | 1 | .51 |
| Anhedonic depression | .13 | 1 | .72 |
| Group × Choice outcome | 1.87 | 2 | .39 |
| Group × Self-critical rumination | .19 | 1 | .67 |
| Choice outcome × Self-critical rumination | 3.58 | 2 | .17 |
| Group × Choice outcome × Self-critical rumination | 9.72 | 2 | .01 |

| **Table S11. Cardiovascular reactivity** | | | | | | |
| --- | --- | --- | --- | --- | --- | --- |
| Variable | *Sum of Squares* | *Mean Square* | *df_Num_* | *df_Den_* | *F* | *p* |
| Group | 0 | 0 | 1 | 77 | .01 | .99 |
| Choice outcome | 162561 | 81280 | 2 | 63862 | 2006.50 | <.001 |
| Time | 60246 | 8607 | 7 | 63828 | 212.47 | <.001 |
| Self-critical rumination | 2 | 2 | 1 | 77 | .05 | .93 |
| Anhedonic depression | 18 | 18 | 1 | 75 | .46 | .50 |
| Group × Choice outcome | 263 | 131 | 2 | 63863 | 3.24 | .03 |
| Group × Time | 204 | 29 | 7 | 63828 | .71 | .66 |
| Choice outcome × Time | 10388 | 742 | 14 | 63828 | 18.32 | <.001 |
| Group × Self-critical rumination | 4 | 4 | 1 | 77 | .09 | .76 |
| Choice outcome × Self-critical rumination | 373 | 187 | 2 | 63866 | 4.61 | .01 |
| Time × Self-critical rumination | 591 | 84 | 7 | 63828 | 2.08 | .04 |
| Group × Choice outcome × Time | 95 | 7 | 14 | 63828 | .17 | .99 |
| Group × Choice outcome × Self-critical rumination | 292 | 146 | 2 | 63866 | 3.60 | .03 |
| Group × Time × Self-critical rumination | 873 | 125 | 7 | 63828 | 3.08 | .003 |
| Choice outcome × Time × Self-critical rumination | 165 | 12 | 14 | 63828 | .29 | .99 |
| Group × Choice outcome × Time × Self-critical rumination | 629 | 45 | 14 | 63828 | 1.11 | .34 |
| *Notes*. *df_Num_* indicates degrees of freedom numerator; *df_Den_* indicates degrees of freedom denominator. | | | | | | |

**Post-hoc Power Analyses**

A series of post-hoc power analyses for every observed tDCS effect in which self-critical rumination was implied (i.e., self-reported counterfactual thinking, self-reported regret, skin conductance responses, cardiovascular reactivity), was carried out using the SIMR r-package^9^. In these post-hoc power analyses, for each effect, the inputted effect size was 90 % of the size of the actual observed effect size, and the power analysis was based on 200 simulations. For the *group* × *self-critical rumination* effect on self-reported counterfactual thinking, 85.50 % power was estimated, 95 % CI [79.84, 90.07]. For the *group* × *self-critical rumination* effect on self-reported regret, 42.50 % power was estimated, 95 % CI [35.56, 49.67]. For the *group* × *choice outcome* × *self-critical rumination* effect on skin conductance responses, 72.00 % power was estimated, 95 % CI [65.23, 78.10]. For the *group* × *choice outcome* × *self-critical rumination* effect on cardiovascular reactivity, 64.50 % power was estimated, 95 % CI [57.44, 71.12].

**References**

1 Schwartz, B. *et al.* Maximizing versus satisficing: Happiness is a matter of choice. *J. Pers. Soc. Psychol.* **83**, 1178-1197, doi:<http://dx.doi.org/10.1037/0022-3514.83.5.1178> (2002).

2 Carver, C. S. & White, T. L. Behavioral-Inhibition, Behavioral Activation, and Affective Responses to Impending Reward and Punishment - the Bis Bas Scales. *J. Pers. Soc. Psychol.* **67**, 319-333, doi:<http://dx.doi.org/10.1037/0022-3514.67.2.319> (1994).

3 Wardenaar, K. J. *et al.* Development and validation of a 30-item short adaptation of the Mood and Anxiety Symptoms Questionnaire (MASQ). *Psychiatry Res* **179**, 101-106, doi:10.1016/j.psychres.2009.03.005 (2010).

4 Smart, L. M., Peters, J. R. & Baer, R. A. Development and Validation of a Measure of Self-Critical Rumination. *Assessment* **23**, 321-332, doi:10.1177/1073191115573300 (2016).

5 Gross, J. J. & John, O. P. Individual differences in two emotion regulation processes: implications for affect, relationships, and well-being. *J. Pers. Soc. Psychol.* **85**, 348 (2003).

6 Garnefski, N. & Kraaij, V. The cognitive emotion regulation questionnaire. *European Journal of Psychological Assessment* **23**, 141-149 (2007).

7 Braithwaite, J. J., Watson, D. G., Jones, R. & Rowe, M. A guide for analysing electrodermal activity (EDA) & skin conductance responses (SCRs) for psychological experiments. *Psychophysiology* **49**, 1017-1034 (2013).

8 Lo, S. & Andrews, S. To transform or not to transform: using generalized linear mixed models to analyse reaction time data. *Front. Psychol.* **6**, doi:10.3389/fpsyg.2015.01171 (2015).

9 Green, P. & MacLeod, C. J. SIMR: an R package for power analysis of generalized linear mixed models by simulation. *Methods in Ecology and Evolution* **7**, 493-498, doi:<https://doi.org/10.1111/2041-210X.12504> (2016).
